# Synthesis and Antileishmanial Activity of 1,2,4,5-Tetraoxanes against *Leishmania donovani*

**DOI:** 10.3390/molecules25030465

**Published:** 2020-01-22

**Authors:** Lília I. L. Cabral, Sébastien Pomel, Sandrine Cojean, Patrícia S. M. Amado, Philippe M. Loiseau, Maria L. S. Cristiano

**Affiliations:** 1 Center of Marine Sciences, CCMAR, Gambelas Campus, University of Algarve, UAlg, 8005-139 Faro, Portugal; liliacabral80@gmail.com (L.I.L.C.); patricia.s.amado@gmail.com (P.S.M.A.); 2 Department of Chemistry and Pharmacy, Faculty of Sciences and Technology, FCT, Gambelas Campus, University of Algarve, UAlg, 8005-139 Faro, Portugal; 3 Chimiothérapie Antiparasitaire, Université Paris-Saclay, CNRS, BioCIS, 92290 Châtenay-Malabry, France; sebastien.pomel@u-psud.fr (S.P.); sandrine.cojean@u-psud.fr (S.C.)

**Keywords:** Leishmaniasis, antileishmanial chemotherapy, peroxide-derived antimalarials, tetraoxanes, antimalarials re-purposing

## Abstract

A chemically diverse range of novel tetraoxanes was synthesized and evaluated in vitro against intramacrophage amastigote forms of *Leishmania donovani*. All 15 tested tetraoxanes displayed activity, with IC_50_ values ranging from 2 to 45 µm. The most active tetraoxane, compound LC140, exhibited an IC_50_ value of 2.52 ± 0.65 µm on *L. donovani* intramacrophage amastigotes, with a selectivity index of 13.5. This compound reduced the liver parasite burden of *L. donovani*-infected mice by 37% after an intraperitoneal treatment at 10 mg/kg/day for five consecutive days, whereas miltefosine, an antileishmanial drug in use, reduced it by 66%. These results provide a relevant basis for the development of further tetraoxanes as effective, safe, and cheap drugs against leishmaniasis.

## 1. Introduction

Leishmaniases are neglected diseases caused by protozoan parasites of the genus *Leishmania* and transmitted by the bite of plebotomine sandflies. Visceral leishmaniasis, the most virulent among leishmaniases, affects mostly tropical and subtropical areas of the world. However, it is spreading out of these areas, namely along southern Europe [1,2]. As with other neglected and poverty-related diseases, most patients suffering from leishmaniases do not benefit from a complete treatment, due to the high cost of available drugs, the need for a long treatment period, low accessibility, an inadequate mode of administration, and drug resistance [3]. These drawbacks have triggered a search for new treatment methods, preferably based on recent technologies. The novel drugs for leishmaniasis should be potent and effective, able to clear the parasite burden in a few days, active against resistant strains of *Leishmania donovani*, orally available, safe, and affordable by the standards of the affected populations [4].

The increasing use of artemisinin and derivatives has clearly evidenced the potential of peroxides in the treatment of vector-borne diseases [5]. Artemisinins were found to be active against all strains of *Plasmodium sp*. and have been used as antimalarials for around three decades, mostly in Artemisinin Combination Chemotherapy (ACT) protocols [6]. However, the high cost of artemisinin, associated with the low yield of extraction from its natural source (*Artemisia annua*), together with some toxicity and a short plasma half-life, leading to complex administration regimens or recrudescence, restricts the therapeutic potential of artemisinins. In addition, recent findings of decreased clinical efficacy of ACTs in Southeast Asia due to resistance [7] have raised concerns over the lifetime of this class as antimalarials. In order to overcome these limitations while maintaining efficacy, various synthetic analogues, incorporating the key peroxide pharmacophore of artemisinin, were developed [8,9]. Among these, trioxolanes and tetraoxanes have shown activity against different parasites, such as the protozoans *Plasmodium spp.* [10,11,12,13,14,15,16,17,18,19], *Perkinsus spp.* [20], and the parasitic flatworms *Schistosoma spp* [21]. A main advantage of trioxolanes and tetraoxanes is their availability, due to straightforward synthesis from inexpensive starting materials, enabling the preparation of chemically diverse libraries of analogues and a better selection of a lead compound [22,23].

The use of artemisinin and its semi-synthetic derivatives for the treatment of leishmaniases has been proposed by several authors [24,25,26,27,28,29,30,31,32,33]. Regarding the potential application of synthetic endoperoxides with antimalarial properties, Cortes et al. [34] reported the antiparasitic activity of a small selection of trioxolanes against promastigote and intramacrophage amastigote forms of *Leishmania infantum*, at micromolar concentrations, introducing the relevance of synthetic endoperoxides for antileishmanial chemotherapy. Given this observation of the antileishmanial activity of ozonide-type antimalarials, it seemed logical to explore 1,2,4,5-tetraoxanes, which also incorporate the endoperoxide core, although these compounds exhibit an enhanced thermodynamic stability compared with their 1,2,4-trioxolane [35,36] or 1,2,4-trioxane [37] counterparts. This singular thermodynamic stability observed in 1,2,4,5-tetraoxanes was clarified by Gomes et al. [38] through theoretical calculations based on stereoelectronic analysis, where the enhanced stability was attributed to a stereoelectronic ‘double anomeric effect’ that stabilizes the six-membered ring system.

Therefore, our aim was to synthesize 1,2,4,5-tetraoxanes, analogues of the ozonides already reported to have antileishmanial activity [31]. For comparison, we have also prepared novel unsymmetrical 1,2,4,5-tetraoxanes and 1,2,4-trioxolanes with polar water-solubilizing groups (Table 1), known to reduce neurotoxicity and increase the activity profiles, as reported in previous works based on artemisinin derivatives [39]. In the present contribution, we disclose the low micromolar activity of a range of peroxides comprising 15 tetraoxanes and two trioxolanes against intramacrophage amastigote forms of *L. donovani*. The results are compared with those of dihydroartemisinin (DHA), artesunate (ATS), and the antileishmanial drug miltefosine. From the tested tetraoxanes, compound LC140 displayed a slight in vivo activity against *L. donovani*. It is worth noting that 1,2,4,5-tetraoxanes are easily prepared, offering the possibility of new candidates with improved pharmacologic profiles.

## 2. Results and Discussion

All tested peroxides showed antiproliferative activity against intramacrophage amastigote forms of *L. donovani*, exhibiting IC_50_ values in a range from 2 to 45 µm and clearly demonstrating the susceptibility of *L. donovani* parasites to the peroxide chemotype (Table 1, entries A–T). Overall, the values are higher than those obtained for miltefosine (0.71 ± 0.20 µm, Table 1, entry T), using the same parasite strain and similar experimental conditions, but the tetraoxanes appeared generally to be less toxic. Among the tested tetraoxanes, three compounds exhibited an IC_50_ value lower than 10 µm (compounds LC140, LC137, and LC165; 2.52 ± 0.65, 7.75 ± 1.12, and 8.79 ± 1.79 μm, respectively; Table 1, entries G, E, and M), LC165 being significantly less toxic than miltefosine. Interestingly, compounds LC137 and LC140 may be obtained from commercially available materials with only two synthetic steps.

Our results indicate that the activities shown by tetraoxanes and trioxolanes with a close chemical structure are similar. For the two tested trioxolanes, LC129 and LC136, the IC_50_ values ranged between 16.30 ± 2.41 and 18.36 ± 4.97 µm (Table 1; entries C, D). These values are similar to those obtained for tetraoxanes LC163, LC177, and PA5 (Table 1; entries L, O, and R). The IC_50_ value obtained for tetraoxane LC165 is also very close to that previously reported for the corresponding trioxolane [34].

From our results, it is possible to conclude that the chemical nature of the cyclohexyl substituent has an impact on activity, for both tetraoxanes and trioxolanes. However, due to the relatively narrow range of values, differences are possibly related to variations in ClogP, with different drug uptakes and pharmacokinetics. Quite interestingly, the activities of all of the artemisinin-derived compounds assayed also lie in the low micromolar range. DHA and tetraoxane LC140 exhibited similar IC_50_ values (3.07 ± 0.45 µm and 2.52 ± 0.65 µm), respectively (Table 1; entry A and Table 1; entry G), while the more polar ATS was shown to be slightly less active (15.00 ± 0.63 µm (Table 1; entry B)).

Our results showed that the activities exhibited by the synthetic tetraoxanes LC137, LC140, and LC165 are similar to those of the semisynthetic artemisinin derivatives (DHA and ATS), disclosing the potential of tetraoxanes to be anti-proliferative agents against intramacrophage amastigote forms of *L. donovani.* The peroxide bridge in the synthetic compounds should play a role in the mechanism of action, as seen for artemisinin and its semisynthetic derivatives. It has been observed that artemisinin mediates its toxicity against *Leishmania* promastigotes by inducing a redox imbalance following the generation of reactive oxygen species (ROS) secondary to cleavage of its endoperoxide bridge, the process terminating in a caspase-independent, apoptotic mode of cell death [25,28,29,33]. It is important to highlight that 1,2,4,5-tetraoxanes have been reported to possess a higher stability and better antimalarial activity compared to their ozonide counterparts [37]. In this work, we can observe that both classes of endoperoxides exhibit similar anti-leishmanial activities, though better IC_50_ values in tetraoxanes LC137, LC140, and LC165 were observed. Future studies for comparison of metabolic properties should be considered.

Concerning the in vivo antileishmanial evaluation, the treatment regimen at a dose of 10 mg/kg/day, for five consecutive days, corresponds to the classical flowchart used by Drugs for Neglected Diseases Initiative (DNDi), the non-governmental organization (NGO) in charge of drug development against Neglected Diseases. DNDi considers further development for a compound only if a significant activity can be demonstrated under these stringent conditions.

**Figure 1:**
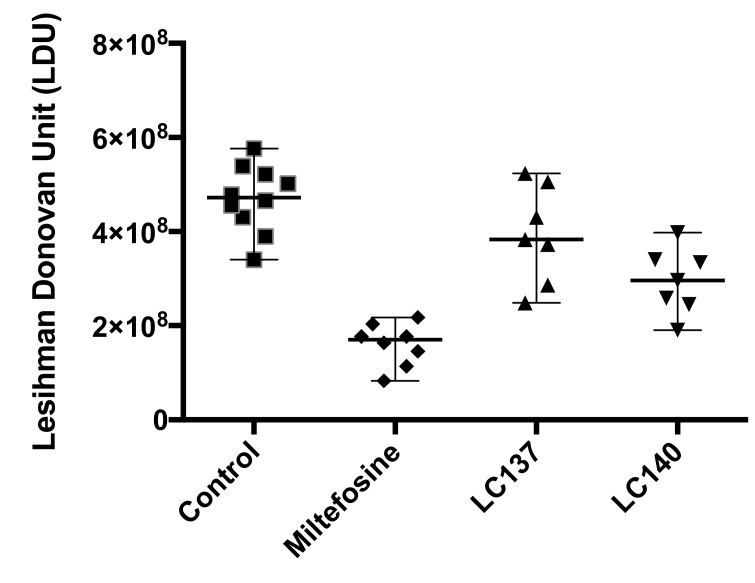
In vivo activity of tetraoxanes LC137 and LC140, and miltefosine, a reference antileishmanial drug.

In vivo, we observed that one mouse among eight mice died in each treated batch (LC137 and LC140) one day after the last treatment. This datum is in relation to a toxicity, whereas no other apparent signs of toxicity were observed (Figure 1). At this early stage, no deeper investigation was performed at the toxicological level. Under these conditions, only the in vivo activity of miltefosine was statistically significant, with a reduction of 66% of the parasite burden in the liver, whereas compound LC140 reduced the liver parasite burden by 37% (Table 2). These results justify further pharmacomodulations in order to optimize this series and to obtain a better in vivo effect at 10 mg/kg/day for five consecutive days, these regimen conditions being sine qua non to go further.

## 3. Materials and Methods

The structures of the 15 tetraoxanes assayed against intramacrophage amastigote forms of *L. donovani* differ only in the chemical nature of the cyclohexyl substituent (Table 1). From this library, only compounds L137 [40], LC140 [14], L153 [41], and LC163 [19] were previously reported in the context of antiparasitic chemotherapy (compound LC163 was disclosed by our group). For comparative purposes, we have also evaluated the activity of a small library of 1,2,4-trioxolanes and that of the known peroxide-based antiplasmodial drugs dihydroartemisinin (DHA) and artesunate (ATS) (Table 1). The 1,2,4,5-tetraoxanes and 1,2,4-trioxolanes were synthesized by adapting procedures described in the literature. The synthetic procedures and experimental details for the preparation and chemical characterization of compounds are included in the Supplementary Materials. The inhibitory effect of each compound tested against *L. donovani* is expressed as IC_50_ (concentration of drug inhibiting parasite growth by 50%), according to a protocol previously described [42]. The cytotoxicity was evaluated on the mouse monocyte/macrophage cell line RAW 264.7, as the parasite host cells, and is expressed as CC_50_ (cytotoxic concentration inhibiting the cell growth by 50%) following a previously described protocol [42]. The selectivity index (SI) is defined as the ratio CC_50_/IC_50_. Miltefosine was used as reference drug. Results are compiled in Table 1.

For in vivo evaluation, all procedures involving animals were conducted in compliance with the standards for animal experiments and were approved by the local committee for animal care (0858.01/2014, Versailles, France). The protocol of evaluation on the *L. donovani*/Balb/C mice model is presented by Morais et al. [43] Two 1,2,4,5-tetraoxane derivatives were evaluated by an intraperitoneal route at 10 mg/kg/day on five consecutive days. Miltefosine, as the control, was evaluated at the same dose by an intravenous route. Animals were sacrificed three days after the end of treatment. Livers and spleens were weighed and drug activity was estimated microscopically by counting the number of amastigotes/500 liver cells in Giemsa-stained impression smears to calculate the *Leishmania donovani* units (LDUs) for liver parasite burdens, using Stauber’s formula. The mean number of parasites per gram of liver among treatment groups and controls was compared. Three independent counts were performed and the results are expressed as the mean values ± SD. The parasite burden of treatment groups and controls were compared using the Kruskal-Wallis nonparametric analysis of variance test for comparing two groups. Significance was established for a *p* value <0.05.

## 4. Conclusions

The results presented herein unveil the potential of tetraoxanes as anti-proliferative agents against intramacrophage amastigote forms of *L. donovani*. Compounds LC137, LC140, and LC165 (Table 1) appear to be the most promising, combining a comparatively high activity and low toxicity. In vivo, LC140 appears to be a lead to investigate further through new pharmacomodulations (see Table 2).

Our data indicate that tetraoxanes and trioxolanes with a close chemical structure exhibit similar activity. Also, the nature of the substituents attached to the endoperoxide core (tetraoxane or trioxolane) appears to have a relatively modest effect on activity. Major aspects (accumulation, bioactivation, targets involved, etc.) underlining the action of the tested compounds (including artemisinin derivatives) require a deep and pluridisciplinary investigation, to unravel the mode of action of these compounds.

## Figures and Tables

**Table 1:** Inhibitory concentrations (IC_50_) of artemisinin derivatives, synthetic 1,2,4-trioxolanes, 1,2,4,5-tetraoxanes, and miltefosine (control) against intramacrophage amastigote forms of *Leishmania* *donovani* LV9, evaluation of cytotoxicity (CC_50_) against the macrophage cell line RAW 264.7, selectivity index (SI), and estimated ClogP values.

Entry	Compounds	Structures	Activity IC_50_ ± SD (µm)	Toxicity CC_50_ ± SD (µm)	SI	ClogP value ^a^
(A)	DHA	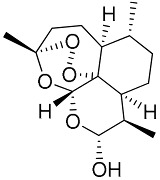	3.07 ± 0.45	>75.00	>24.00	2.59
(B)	ATS	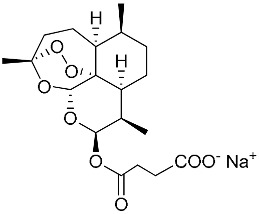	15.00 ± 0.63	>75.00	5.00	2.68
(C)	LC129	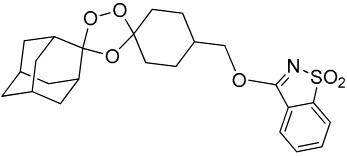	16.30 ± 2.41	>75.00	>4.00	4.66
(D)	LC136	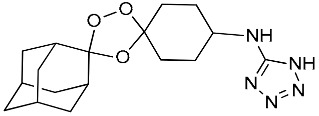	18.36 ± 4.97	55.20 ± 6.30	3.00	3.14
(E)	LC137	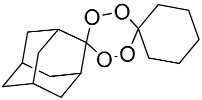	7.75 ± 1.12	43.15 ± 3.25	5.50	4.52
(F)	LC139	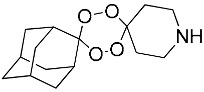	13.17 ± 0.03	>50.00	>3.80	2.91
(G)	LC140	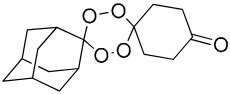	2.52 ± 0.65	34.12 ± 5.38	13.50	3.19
(H)	LC141	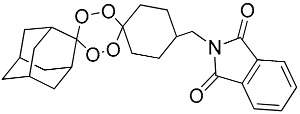	18.36 ± 3.19	>100.00	5.40	5.24
(I)	LC146	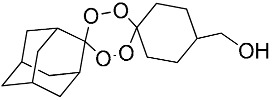	16.00 ± 1.05	>100.00	>6.20	3.59
(J)	LC153	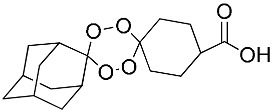	17.33 ± 2.02	>50.00	>2.80	3.64
(K)	LC159	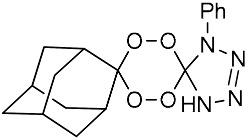	44.49 ± 1.13	ND	ND	4.39
(L)	LC163	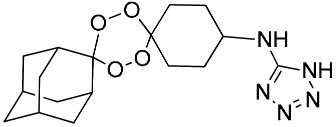	12.16 ± 3.96	>50.00	>4.10	3.41
(M)	LC165	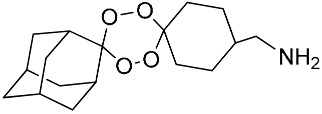	8.79 ± 1.79	>75.00	>8.00	3.35
(N)	LC167	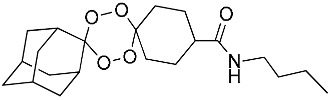	14.97 ± 0.07	>50.00	>3.30	4.67
(O)	LC177	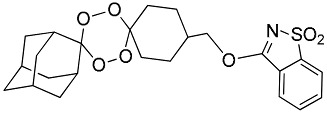	17.57 ± 0.85	>50.00	>2.80	4.89
(P)	LC179	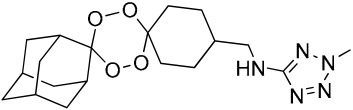	29.05 ± 0.26	ND	ND	4.16
(Q)	LC180	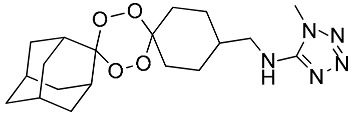	28.97 ± 1.95	ND	ND	3.76
(R)	PA5	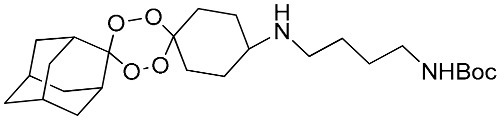	19.22 ± 0.11	>50.00	>2.60	4.83
(S)	PA6	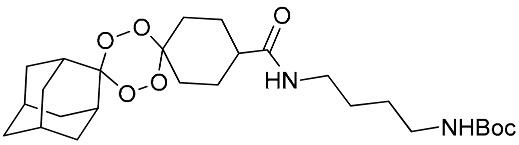	17.74 ± 2.78	>50.00	>2.80	4.53
(T)	HePC (Miltefosine)	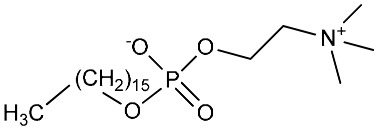	0.71 ± 0.20	54.30 ± 2.14	76.40	4.22

ND—not determined. ^a^ Calculated using ALOGPS software (http://www.vcclab.org/lab/alogps/).

**Table 2:** In vivo activity of tetraoxanes LC137 and LC140, and miltefosine, a reference antileishmanial drug, on *L. donovani*/Balb/C mice. Compounds were administered intraperitonially, at a dose of 10 mg/kg/day, for five consecutive days.

Batch	Number of Mice	Number of Dead Mice	Treatment Regimen	LDU (10^8^) ± SD	% reduction of Parasite Burden in the Liver
LC137 10 mg/kg	8	1	10 mg/kg/d x5 (IP)	3.92 ± 1.03	16.40
LC140 10 mg/kg	8	1	10 mg/kg/d x5 (IP)	2.95 ± 0.69	37.29
Miltefosine	8	0	10 mg/kg/d x5 (IV)	1.60 ± 0.45	65.9
Control	10	0	Treated with the excipient	4.70 ± 0.71	-

## Supplementary Materials

The following are available online, S.1: Synthetic procedures and experimental details for the synthesis and chemical characterization of compounds. S.1.1: General methods and analytical techniques. S.1.2: Preparation of intermediate building blocks. S.1.2.1: Preparation of 3-chloro-1,2-benzisothiazole-1,1-dioxide. S.1.2.2: Preparation of 1-phenyl-*1H*-tetrazol-5(*4H*)-one, 1-methyl-*1H*-tetrazole-5-amine and 2-methyl-*2H*-tetrazole-5-amine. S.1.2.3: Preparation of tert-butyl(4-aminobutyl)carbamate, LC64. S.1.3: Synthetic route to trixolanes. S.1.3.1: Synthesis of 1,2,4-trioxolanes LC129 and LC136. S.1.4: Synthetic route to tetraoxanes. S.1.4.1: Synthesis of 1,2,4,5-tetraoxanes. S.2: Spectra of the compounds. S3. In vitro antileishmanial screening. S3.1: Cell lines and cultures. S3.2: In vitro antileishmanial evaluation on intramacrophage amastigotes. S3.3: In vitro antileishmanial evaluation on *Leishmania donovani* axenic amastigotes. S3.4: Evaluation of compounds cytotoxicity. S4. In vivo antileishmanial screening. S4.1: Animal and housing. S4.2: Evaluation of in vivo acute toxicity by an intraperitoneal route. S4.3: In vivo antileishmanial evaluation. S.5: References.
