# Supplementary material for: Synthesis and Antileishmanial Activity of 1,2,4,5-Tetraoxanes against Leishmania donovani
Source: Molecules. 2020 Jan 22;25(3):465. doi: 10.3390/molecules25030465 (PMC7038143; doi:10.3390/molecules25030465)
Supplement: Supplementary file 1 [file molecules-25-00465-s001.pdf]

Supplementary material

Synthesis and antileishmanial activity of 1,2,4,5-tetraoxanes against *Leishmania donovani*

Lília I. L. Cabral[1,2], Sébastien Pomel[3], Sandrine Cojean[3], Patrícia S. M. Amado[1,2], Philippe M. Loiseau[3,]*, Maria L. S. Cristiano[1,2,]*

[1] Center of Marine Sciences, CCMAR, Gambelas Campus, University of Algarve, UAlg, Portugal; liliacabral80@gmail.com (L.I.L.C.) a41651@ualg.pt (P.S.M.A.); mcristi@ualg.pt (M.L.S.C.)

[2] Department of Chemistry and Pharmacy, Faculty of Sciences and Technology, FCT, Gambelas Campus, University of Algarve, UAlg, Portugal

[3] Chimiothérapie Antiparasitaire, Université Paris-Saclay, CNRS, BioCIS, 92290 Châtenay-Malabry, France; Sébastien.pomel@u-psud.fr (S.P.); sandrine.cojean@u-psud.fr (S.C.); philippe.loiseau@u-psud.fr (P.L.)

* Correspondence: mcristi@ualg.pt, Tel.: +351-912-074-576 (M.L.S.C); philippe.loiseau@u-psud.fr (P.L.)

S.1. Synthetic procedures and experimental details for the synthesis and chemical characterization of compounds. 2
S.1.1. General methods and analytical techniques 2
S.1.2. Preparation of intermediate building blocks 2
S.1.2.1. Preparation of 3-chloro-1,2-benzisothiazole-1,1-dioxide 2
S.1.2.2. Preparation of 1-phenyl-*1H*-tetrazol-5(*4H*)-one, 1-methyl-*1H*-tetrazole-5-amine and 2-methyl-*2H*-tetrazole-5-amine 3
S.1.2.3. Preparation of tert-butyl(4-aminobutyl)carbamate, LC64 4
S.1.3. Synthetic route to trioxolanes 5
S.1.3.1. Synthesis of 1,2,4-trioxolanes LC129 and LC136 5
2-(Methoxyimino)adamantane, LC29 5
S.1.4. Synthetic route to tetraoxanes 8
S.1.4.1. Synthesis of 1,2,4,5-tetraoxanes 10
S.2. Spectra of the compounds 17
S3. *In vitro* antileishmanial Screening 37
S3.1. Cell lines and cultures. 37
S3.2. *In vitro* antileishmanial evaluation on intramacrophage amastigotes. 37
S3.3. *In vitro* antileishmanial evaluation on *Leishmania donovani* axenic amastigotes 38
S3.4. Evaluation of compounds cytotoxicity. 38
S4. *In vivo* antileishmanial Screening 40
S4.1. Animal and housing 40

S4.2. Evaluation of *in vivo* acute toxicity by intraperitoneal route. 40
S4.3. *In vivo* antileishmanial evaluation 41
S.5. References...42

S.1. Synthetic procedures and experimental details for the synthesis and chemical characterization of compounds.

S.1.1. General methods and analytical techniques

Commercial reagents were used as purchased. When required, solvents were dried following standard procedures.[1] ^{1}H and ^{13}C-NMR spectra were recorded on a 400 MHz NMR spectrometer Bruker Avance III 400. ^{1}H-NMR-chemical shifts are referred to the residual signal of $CDCl_3$ (δH 7.27) and ^{13}C-NMR- chemical shifts to the $CDCl_3$ signal (δC 77.0), or using TMS as internal standard. Thin-layer chromatography was carried out on silica gel 60 F254 plates (AL TLC 20x20). Column chromatography was performed on Silica Gel 60 (0.04 – 0.063 mm). IR spectra were recorded on a Tensor 27 FT/IR spectrometer in the 600–3800 cm^{-1} range. Melting points (℃) were obtained on a SMP3 Melting Point Apparattus and are uncorrected.

S.1.2. Preparation of intermediate building blocks

The synthetic approach followed to the preparation of intermediate blocks for the synthesis of final target compounds is illustrated in Schemes S3-S5. Synthetic procedures for each compound prepared are also provided in this section.

S.1.2.1. Preparation of 3-chloro-1,2-benzisothiazole-1,1-dioxide

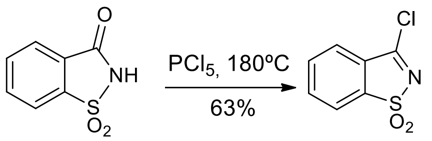

LC60

Scheme S3

The experimental procedure used has been reported previously.[2] Starting from saccharin (56 mmol) and phosphorus pentachloride (66 mmol), heated at 200 ℃. Colourless needles from ethanol (63% yield); m.p. 143-145 ℃. IR ν_{max} (cm^{-1}): 1724, 1654, 1603 (C=C), 1346 (SO_2),

775 (Ar-H) and 692 (C-Cl); ^{1}H-NMR (400 MHz, $CDCl_3$): δ 7.85 (4H, m, Ar-H) ppm. Found: C, 41.5%; H, 2.0%; N, 6.9%; calcd for $C_7H_4NO_2SCl$: C, 41.7%; H, 2.0%; N, 7.0%. MS (EI, m/z): 201 $[M]^+$.

S.1.2.2. Preparation of 1-phenyl-*1H*-tetrazol-5(*4H*)-one, 1-methyl-*1H*-tetrazole-5-amine and 2-methyl-*2H*-tetrazole-5-amine

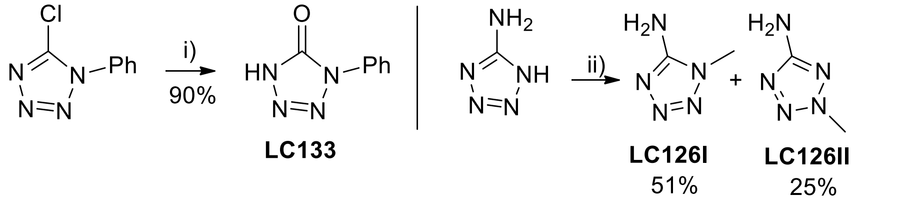

Scheme S4: Reagents and conditions: i) NaOH (5M), r.t.; ii) Dimethylsulfate, NaOH/H_2O, phenolphthalein, 100 ℃.

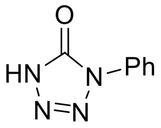

1-phenyl-*1H*-tetrazole-5-one, LC133[3]

5-Chloro-1-phenyl-tetrazole (1 eq) was added to a solution of sodium hydroxide (5M, 10 mL). The reaction mixture was stirred at room temperature for 24 h. The resulting solution was cooled to room temperature and acidified by addition of HCl (aq) (10%; pH≈1). A precipitate was formed, filtered and washed with chloroform and hexane to give the product (90% yield) as a colourless powder; m.p. 97-99 ℃; ^{1}H-NMR (400 MHz, $CDCl_3$): δ 7.57 (m, 2H), 7.66 (s, 1H), 7.70 (d, J = 6.5 Hz, 1H) ppm; MS (EI, *m/z*) : 162,05 $[M]^+$.

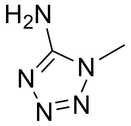

1-methyl-*1H*-tetrazole-5-amine, LC126I[4]

A solution of sodium hydroxide (20%) was added dropwise to a suspension of 5-aminotetrazole monohydrate (120 mmol) in water (30 mL), with a drop of phenolphthalein. The mixture was stirred until complete dissolution of the suspended material. Dimethyl sulphate (110 mmol) was then added in small portions, keeping an alkaline medium through addition of aqueous sodium hydroxide. The final mixture was refluxed for 1 h, then cooled, and finally left in ice bath for 48h. Colourless needles of the desired compound were filtered

and dried (51% yield); m.p. 220-221 ℃. ^{1}H-NMR (400 MHz, $CDCl_3$): δ 4.15 (s, 3H) ppm; MS (EI, *m/z*): 99 $[M]^+$.

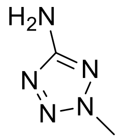

2-methyl-*2H*-tetrazole-5-amine, LC126II[4]

The filtrate from 1-methyl-1H-tetrazole-5-amine **LC126I** synthesis was evaporated under reduced pressure to afford a solid residue. Water (50 mL) was added, and the mixture was then extracted with diethyl ether (3 x 50 mL). The organic extract was dried over anhydrous sodium sulfate, filtered, and the filtrate evaporated to afford colourless crystals. Recrystallization from diethyl ether gave the desired compound as colourless needles (25% yield); m.p. 104.5-105.5 ℃. ^{1}H-NMR (400 MHz, $CDCl_3$): δ 3.32 (s, 3H) ppm; MS (EI, m/z): 99 $[M]^+$.

S.1.2.3. Preparation of tert-butyl(4-aminobutyl)carbamate, LC64[5]

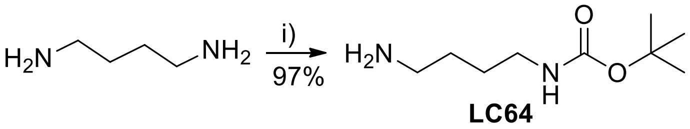

Scheme S5: Reagents and conditions: i) Boc_2O, 1,4-dioxane, r.t.

A solution of di-*tert*-butyl dicarbonate (2.50 x 10^{-2} mol) in 1,4-dioxane (100 mL), under stirring, was added by cannula, over 3 hours, to a stirring solution of 1,4-diaminobutane (1.40 x 10^{-1} mol) in 1,4-dioxane (100 mL). The final reaction mixture was stirred at room temperature for 20 h and then concentrated under reduced pressure. Water was added to precipitate the formed conjugate. The aqueous residue was extracted with DCM (2 x 30 mL). The combined organic extracts were dried over anhydrous $MgSO_4$, filtered, and the filtrate was evaporated to dryness under reduced pressure to give a clear oil (97 % yield). ^{1}H-NMR (400 MHz, $CDCl_3$): δ 1.42 (s, 9H), 1.48 (d, J = 7.0 Hz, 4H), 2.71 (s, 2H), 3.10 (s, 2H) ppm; MS (MALDI-TOF, *m/z*): 189,17 $[M+H]^+$.

S.1.3. Synthetic route to trioxolanes

The synthetic approach followed to trioxolanes is illustrated in Scheme S6. Synthetic procedures for the preparation of each compound are also provided in this section (as described in literature[6]).

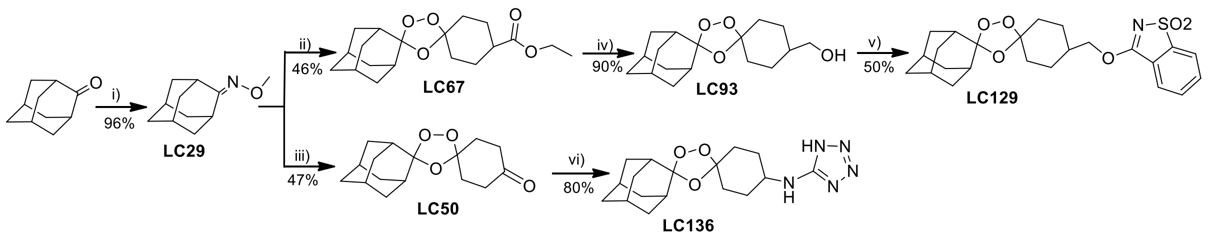

Scheme S6: Reagents and conditions: i) Pyridine, $MeONH_2$, MeOH, r.t; ii) Ethyl 4-oxocyclohexanecarboxylate, O_3, DCM/Pentane, -78 ℃; iii) 1,4-Cyclohexane, O_3, DCM/Pentane, -78 ℃; iv) $LiBH_4$, Et_2O, $LiBH(Et)_3$, r.t.; v) 3-Chloro-1,2-benzisothiazole-1,1-dioxide, TEA, Toluene, 45 ℃; vi) 5-Aminotetrazole monohydrate, AcOH, DCE, $NaBH(OAc)_3$, r.t.

S.1.3.1. Synthesis of 1,2,4-trioxolanes LC129 and LC136

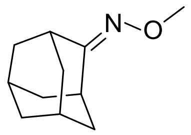

2-(Methoxyimino)adamantane, LC29.[5]
To a solution of 2-adamantanone (30 mmol) in methanol (30 mL) were added pyridine (55.6 mmol) and methoxylamine hydrochloride (45.0 mmol). The reaction mixture was stirred at room temperature for 48 h. The final mixture was concentrated and then diluted with DCM (50 mL) and water (50 mL). The organic layer was separated and the aqueous layer was extracted with DCM (30 mL). The combined organic extracts were washed with aqueous HCl (1 M; 30 mL x2), then with saturated aqueous NaCl (30 mL). The final organic extract was dried over $MgSO_4$, filtered and concentrated under reduced pressure to give O-methyl-2-adamantanone oxime (89% yield) as a colourless solid. m.p. 69-70 ℃ (Lit.[4] 70-71 ℃). ^1H-NMR (400 MHz, $CDCl_3$): δ 1.78-1.97 (m, 12H), 2.53 (s, 1H,), 3.45 (s, 1H), 3.81 (s, 3H) ppm; MS (MALDI-TOF, *m/z*): 180.02 $[M + H]^+$.

General procedure 1: Preparation of Adamantyl-1,2,4-trioxolanes LC50 and LC67. Trioxolanes were prepared by coupling O-methyl-2-adamantanone oxime (2) with a cyclohexanone derivative, through ozonolysis.

Ozone, produced with an ozone generator Sander Labor-Ozonizator 301.7 (0.5 L/min O_2, 140 V), was passed through a solution of dichloromethane at –78 ℃ and flushed into a solution of O-methyl ketone oxime and a ketone, in pentane/dichloromethane (6:4) at 0 ℃. After completion, the solution was flushed with nitrogen for 5 min and concentrated under reduced pressure at room temperature to give a crude material that was purified by column chromatography.

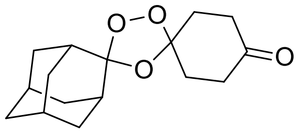

Dispiro[cyclohexane-1,3'-[1,2,4]trioxolane-5',2''-tricyclo[3.3.1.1^{3,7}]decan]-4-one, <u>LC50</u>.[5]

A solution of O-methyl 2-adamantanone oxime (8.4 mmol) and 1,4-cyclohexanedione (11 mmol) in pentane (60 mL) and dichloromethane (40 mL) was treated with ozone (as described in general procedure 1). The crude product was purified by column chromatography (silica gel; ethyl acetate/n-hexane 1/9) to give product **LC50** (42% yield) as a colourless solid; m.p. 127-128 ℃ (Lit.[4] 126-128 ℃). ^{1}H-NMR (400 MHz, $CDCl_3$): δ 1.69-2.02 (m, 14H), 2.14 (t, *J* = 7,1 Hz, 4H), 2.51 (t, *J* = 7,1 Hz, 4H) ppm; ^{13}C-NMR (100 MHz, $CDCl_3$): 25.9, 26.31, 31.09, 32.59, 34.25, 35.70, 36.18, 37.35, 106.46, 111.95, 208.90 ppm; MS (EI, *m/z*): 278.9 [M + H]$^+$.

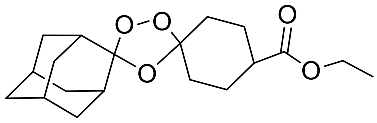

Ethyl dispiro[cyclohexane-1,3'-[1,2,4]trioxolane-5',2''-tricyclo[3.3.1.1^{3,7}]decane]-4-carboxylate, <u>LC67</u>.

A solution of O-methyl 2-adamantanone oxime (20 mmol) and ethyl 4-oxocyclohexanecarboxylate (20 mmol), in pentane (60 mL) and DCM (40 mL), was treated with ozone, according the procedure general 1. The crude product was purified by column chromatography (silica gel, ethyl acetate/n-hexane 1/9) to afford trioxolane **LC67** as a colourless oil (46% yield). ^{1}H-NMR (400 MHz, $CDCl_3$): δ 1.26 (t, *J* = 7,2 Hz, 3H), 1.70-1.76 (m 11H), 1.92-2.03 (m, 12H,), 2.33 (m, 1H), 4.15 (dd, *J* = 7.1 Hz, *J* = 7.0 Hz, 2H) ppm; MS (MALDI-TOF, *m/z*): 337.34 [M + H]$^+$.

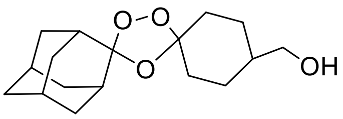

(Dispiro[cyclohexane-1,3'-[1,2,4]trioxolane-5',2''-tricyclo[3.3.1.1^{3,7}]decan]-4-yl)methanol, LC93.

A solution of **LC67** (11.3 mmol), lithium borohydride (11.3 mmol, 2M in THF) and lithium triethylborohydride (1.13 mmol, 1M in THF) in ether (15 mL) was stirred overnight, at room temperature. The reaction mixture was diluted with ether (5 mL), washed with aqueous NaOH (3M; 2 x 10 mL), brine and water (2 x 10 mL). The organic extract was dried over $MgSO_4$, filtered, and concentrated under reduced pressure to give product **LC93** (90% yield) as a a yellow crystalline solid; m.p. 99-101 ℃. ^{1}H-NMR (400 MHz, $CDCl_3$): δ 1.25 (m, 2H), 1.51-2.08 (m, 21H), 3.46 (t, *J* = 7.1 Hz, 2H) ppm; MS (MALDI-TOF, *m/z*): 318.30 [M + Na]$^+$.

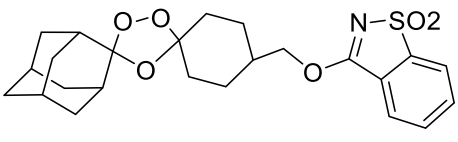

3-{(Dispiro[cyclohexane-1,3'-[1,2,4]trioxolane-5',2''-tricyclo[3.3.1.1^{3,7}]decan]-4-yl)methoxy}-*1H*-1λ^6,2-benzisothiazole-1,1-dione, LC129.[6]

Compound **LC60** (4.08 mmol) was added to a solution of compound **LC93** (3.4 mmol) in dry toluene (30 mL). The solution was stirred at 45 ℃ for 15 minutes, followed by addition of triethylamine (6.8 mmol) until disappearance of all of the starting material. The precipitate of triethylamine hydrochloride was filtered off and the filtrate was evaporated to give a yellow crystalline solid, which was recrystallized from ethanol (50% yield); m.p. 150-151 ℃. ^{1}H-NMR (400 MHz, $CDCl_3$): δ 1.35-1.38 (m, 2H), 1.62-1.96 (m, 21H), 4.36 (d, *J* = 7.2 Hz, 2H), 7.64 (d, *J* = 7.1 Hz, 1H), 7.69 (d, *J* = 7.0 Hz, 2H), 7.82 (d, *J* = 7.2 Hz, 1H) ppm; ^{13}C-NMR (100 MHz, $CDCl_3$): 17.60, 26.87, 29.23, 32.36, 35.39, 37.95, 39.14, 66.39, 108.22, 117.62, 123.30, 127.03, 133.44, 134.12, 143.61, 169.23 ppm. MS (EI, *m/z*): 482.25 [M + Na]$^+$. Anal. calcd for $C_{24}H_{29}NO_6S$: C, 62.73%; H, 6.36%; N, 3.05%; Found: C, 62.85%; H, 6.44%; N, 2.99%.

General procedure 2: Preparation of Adamantyl-1,2,4-trioxolane LC136. The required amine (**LC64** or **5-aminotetrazole**) (3.4 mmol) was added to a solution of compound **LC50** (3.4 mmol) in anhydrous 1,2-dichloroethane (20 mL) and acetic acid (3.4 mmol). The

mixture was allowed to stir at room temperature for 30 minutes, followed by addition of sodium triacetoxyborohydride (8.5 mmol). After stirring at room temperature for 16 hours, the final reaction mixture was washed with aqueous NaOH (5M; 2 x 10 mL) and dichloromethane (2 x 20 mL). The organic extract was dried over $MgSO_4$, filtered, and the solvent evaporated. The crude product was purified by column chromatography (silica gel, ethyl acetate/n-hexane 3/7).

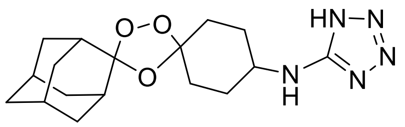

***N*-*1H*-1,2,3,4-Tetraazol-5-yl-dispiro[cyclohexane-1,3'-[1,2,4]trioxolane-5',2''-tricyclo[3.3.1.1^{3,7}]decan]-4-ylamine, <u>LC136</u>.**[6]

Prepared according to general procedure 2 to give **LC136** as a white solid (80% yield); mp 98-100 °C. ^{1}H-NMR (400 MHz, $CDCl_3$): δ 1.24-1.33 (m, 2H), 1.69-1.72 (m, 10H), 1.90-2.05 (m, 10H), 2.66 (m, 1H) ppm; ^{13}C-NMR (100 MHz, $CDCl_3$): 21.05, 27.07, 28.42, 33.67, 36.78, 37.64, 58.57, 117.87, 127.78, 156.15 ppm; MS (MALDI-TOF, *m/z*): 347.31 [M + H]$^+$.

S.1.4. Synthetic route to tetraoxanes

The synthetic approach followed to tetraoxanes is depicted in Schemes S7 to S11. Synthetic procedures (as described in literature[7]) for each compound prepared are also provided in this section.

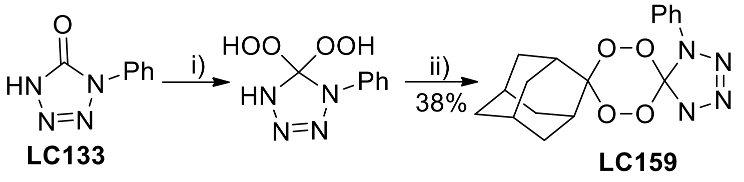

Scheme S7: Reagents and conditions: i) HCO_2H, CH_3N, H_2O_2 50%, 0 °C; ii) Adamantanone, DCM, HBF_4

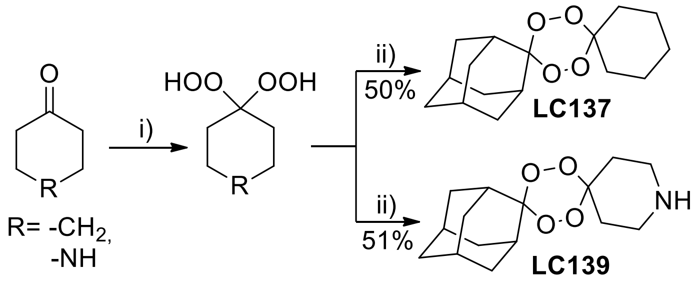

Scheme S8: Reagents and conditions: i) HCO_2H, CH_3CN, H_2O_2 50%, 0 °C; ii) Adamantanone, DCM, HBF_4, 0 °C.

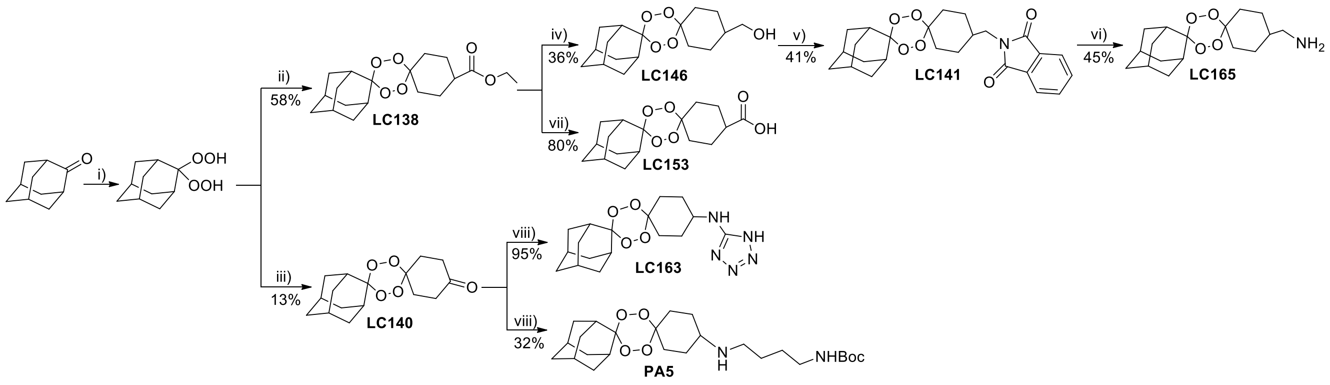

Scheme S9: Reagents and conditions: i) HCO_2H, CH_3CN, H_2O_2 50%, 0 ℃; ii) Ethyl 4-oxocyclohexanecarboxylate **(C)**, DCM, HBF_4, 0 ℃; iii) 1,4-cyclohexanone **(D)**, DCM, HBF_4, 0 ℃; iv) $LiBH_4$, Et_2O, $LiBH(Et)_3$, r.t.; v) Phthalimide, Ph_3P, DIAD, THF, 0 ℃; vi) Hydrazine hydrate, Chloroform/MeOH, 60 ℃; vii) KOH (3M), MeOH, 60 ℃; viii) DCE, AcOH, $NaBH(OAc)_3$, r.t.

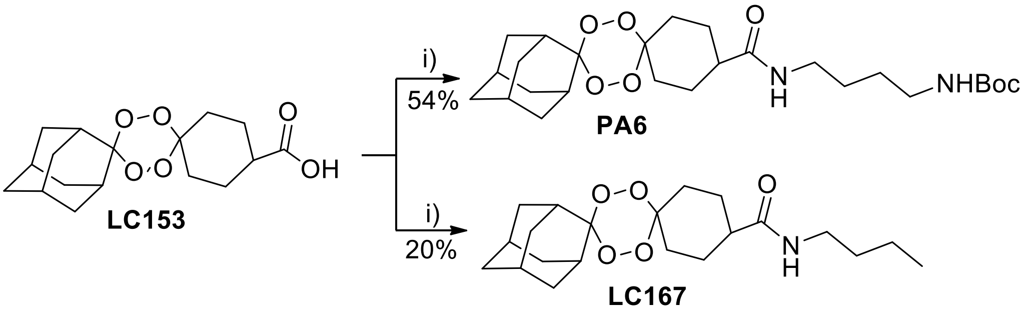

Scheme S10: Reagents and conditions: i) EDC, HOBt, N-methylmorpholine, DCM, r.t.

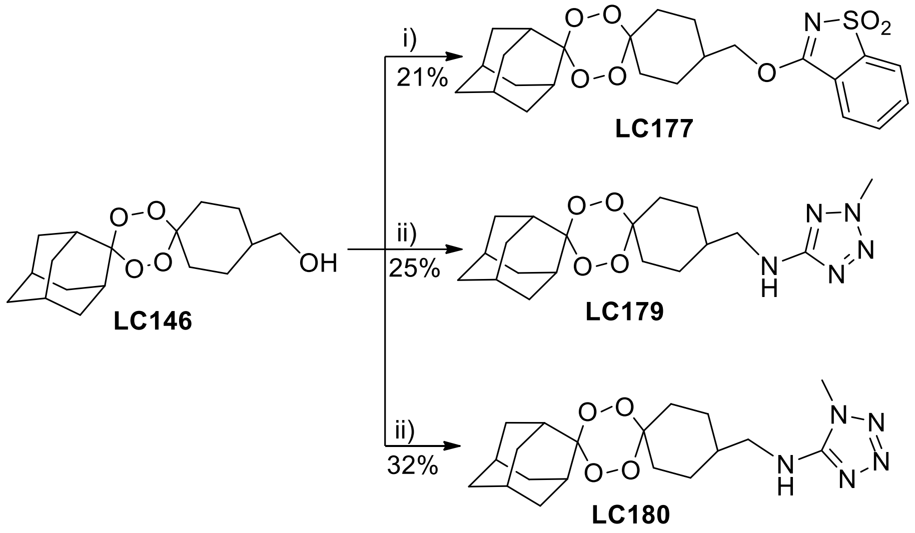

Scheme S11: Reagents and conditions: i) Triethylamine, Toluene, 45 ℃; ii) Triethyamine, mesyl chloride, THF, 60 ℃.

S.1.4.1. Synthesis of 1,2,4,5-tetraoxanes

General Procedure 3: Preparation of Adamantyl-1,2,4,5-tetraoxanes LC137, LC138, LC139, LC140 and LC159. (as described in literature[7]) To a stirring solution of the appropriate ketone A and B, 2-adamantanone or LC133 (5 mmol) in acetonitrile (5.5 mL) and formic acid (3.7 mL) at 0 ℃ was added 50% aq. hydrogen peroxide (1.9 ml). The solution was allowed to warm to room temperature and stirred for 45 min. The solution was diluted with dichloromethane (100 ml) and washed with water (100 ml). The organic phase was dried over Na_2SO_4, filtered and concentrated to give the corresponding gem-dihydroperoxide. A solution of this intermediate in dichloromethane (5 mL) was added to a stirring solution of 2-adamantanone or ketone C and D (7.5 mmol) and 54% ethereal solution of HBF_4 (0.1 mL) in dichloromethane (5 mL) at 0 ℃. The mixture was allowed to warm to room temperature and stirred for 4h. The organic layer was washed with a saturated solution of $NaHCO_3$ and dried over $MgSO_4$ and the solvent removed. The resulting residue was purified by flash column chromatography to give the desired dispiro-1,2,4,5-tetraoxane.

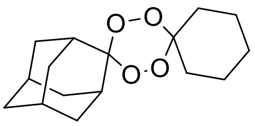

Dispiro[cyclohexane-1,3'-[1,2,4,5]tetraoxane-6',2''-tricyclo[3.3.1.1^{3,7}]decane], LC137.[7]

Prepared according to general procedure 3 to give **LC137** as a white solid (32% yield); m.p. 57-59 ℃ (Lit.[7] 55-58 ℃). ^{1}H-NMR (400 MHz, $CDCl_3$): δ 1.46-1.99 (m, 21H), 2.27 (s, 3H) ppm; ^{13}C-NMR (100 MHz, $CDCl_3$): 25.42, 27.14, 33.23, 37.04, 108.16, 110.13 ppm; MS (MALDI-TOF, *m/z*): 318.33 $[M+K]^+$. Anal. calcd for $C_{16}H_{24}O_4$: C, 68.54%; H, 8.63%; Found: C, 68.49%; H, 8.73%.

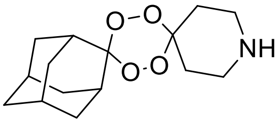

Dispiro[piperidine-4,3'-[1,2,4,5]tetraoxane-6',2''-tricyclo[3.3.1.1^{3,7}]decane], LC139.

Prepared according to general procedure 3 to give **LC139** as a yellow solid (51% yield); m.p. 65-67 ℃. ^{1}H-NMR (400 MHz, $CDCl_3$): δ 1.26 (s, 1H), 1.75 (d, J = 7.3 Hz, 6H), 2.04 (m,

14H), 3.08 (t, *J* = 7.1 Hz, 2H) ppm; ^{13}C-NMR (100 MHz, $CDCl_3$): 27.80, 29.67, 32.67, 33.81, 36.68, 107.39, 109.46 ppm; MS (MALDI-TOF, *m/z*): 282.29 $[M+H]^+$.

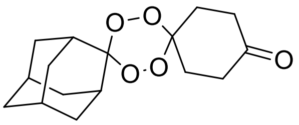

Dispiro[cyclohexane-1,3'-[1,2,4,5]tetraoxane-6',2''-tricyclo[3.3.1.1^{3,7}]decan]-4-one, LC140.[8]

Prepared according to general procedure 3 to give **LC140** as a white solid (13% yield); m.p. 156-158 ℃. ^{1}H NMR (400 MHz, $CDCl_3$): δ 3.19 (br s, 1H), 2.72 (br s, 2H), 2.51 (br s, 4H), 2.11 – 1.91 (m, 7H), 1.90 (br s, 3H), 1.78 – 1.66 (m, 5H) ppm; ^{13}C NMR (101 MHz, $CDCl_3$): δ 209.27, 110.99, 106.59, 36.86, 36.30, 35.55, 34.29, 33.13, 30.23, 30.06, 27.91, 27.45 ppm. MS (MALDI-TOF, *m/z*): 318.28 $[M+Na]^+$.

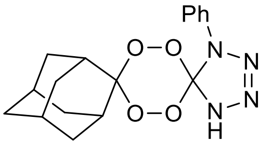

1-Phenyl-*1H,4H*-dispiro[1,2,3,4-tetraazole-5,3'-[1,2,4,5]tetraoxane-6',2''-tricyclo[3.3.1.1^{3,7}]decane], LC159.

Prepared according to general procedure 3 to give **LC159** as a white solid (38% yield); m.p. 200-202 ℃. ^{1}H-NMR (400 MHz, $CDCl_3$): δ 0.83 (m, 2H), 1.18-1.26 (m, 4H), 1.62 (br s, 4H), 1.94-1.96 (m, 5H), 7.46 (dd, *J* = 7.1 Hz, *J* = 7.0 Hz, 2H), 7.64 (dd, *J* = 7.2 Hz, *J* = 7.1 Hz, 2H), 9.40 (s, 1H) ppm; ^{13}C-NMR (100 MHz, $CDCl_3$): 27.75, 32.74, 33.59, 36.69, 110.62, 117.41, 121.34, 130.67, 144.21, 180.67 ppm; MS (MALDI-TOF, *m/z*): 344.33 $[M]^+$.

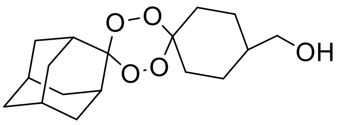

(Dispiro[cyclohexane-1,3'-[1,2,4,5]tetraoxane-6',2''-tricyclo[3.3.1.1^{3,7}]decan]-4-yl)methanol, LC146.

A solution of **LC138** (11.3 mmol), lithium borohydride (11.3 mmol, 2M in THF) and lithium triethylborohydride (1.13 mmol, 1M in THF) in ether (15 mL) was stirred overnight, at rt. The reaction mixture was diluted with ether (5 mL), washed with aqueous NaOH (3M; 2 x 10 mL), brine and water (2 x 10 mL). The organic extract was dried over $MgSO_4$, filtered, and

the solvent removed. The crude product was purified by column chromatography (silica gel, ethyl acetate/n-hexane 2/8) to give product **LC146** as a white solid (36% yield). m.p. 175-177 ℃. ^{1}H-NMR (400 MHz, $CDCl_3$): δ 1.19 (m, 2H), 1.64 (s, 6H), 1.69-1.93 (m, 13H), 2.14 (d, J = 6.7 Hz, 2H), 3.47 (s, 2H) ppm; ^{13}C-NMR (100 MHz, $CDCl_3$): 26.57, 26.78, 27.14, 33.64, 34.91, 34.99, 36.59, 36.98, 39.03, 67.72, 108.68, 111.59 ppm; MS (MALDI-TOF, *m/z*): 309.35 $[M]^-$.

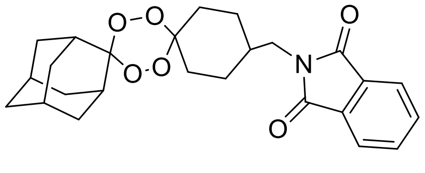

2-{(Dispiro[cyclohexane-1,3'-[1,2,4,5]tetraoxane-6',2''-tricyclo[3.3.1.1^{3,7}]decan]-4-yl)methyl}-1,3-isoindolinedione, LC141.

A solution of **LC146** (9.52 mmol) in dry THF (25 mL) was cooled to 0 ℃. Ph_3P (1.33 mmol), phthalimide (10.5 mmol) and DIAD (1.33 mmol) were gradually added. The mixture was stirred at room temperature for 24 hours. The solvent was then evaporated to dryness and the crude product was purified by column chromatography (silica gel, ethyl acetate/n-hexane 1/9) to give product **LC141** as a yellow crystals (41% yield). m.p. 170-172 ℃. ^{1}H-NMR (400 MHz, $CDCl_3$): δ 1.17-1.29 (m, 2H), 1.80-2.93 (m, 21H), 3.70 (d, J = 7.0 Hz, 2H), 7.52 (m, 1H), 7.59 (m, 1H), 7.71 (m, 2H) ppm; ^{13}C-NMR (100 MHz, $CDCl_3$): 26.54, 27.06, 27.97, 33.75, 34.54, 34.95, 35.64, 36.57, 43.17, 107.52, 111.46, 123.55, 132.45, 133. 97, 168.67 ppm; MS (EI, *m/z*): 439.11 $[M]^+$.

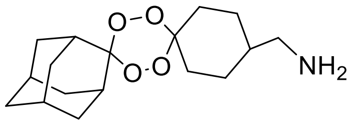

{(Dispiro[cyclohexane-1,3'-[1,2,4,5]tetraoxane-6',2''-tricyclo[3.3.1.1^{3,7}]decan]-4-yl)methyl}amine, LC165.

A solution of **LC141** (7.56 mmol) and hydrazine monohydrate (45.4 mmol) in chloroform and methanol (7:3, 50 mL total) was heated at 60 ℃ for 35 h. The reaction mixture was cooled to room temperature and filtered to remove solid by-products. The filtrate was washed with water (50 mL) and brine (50 mL), dried over $MgSO_4$, filtered, and concentrated to give product **LC165** (45% yield) as light yellow oil. ^{1}H-NMR (400 MHz, $CDCl_3$): δ 1.15-1.38 (m, 2H), 1.62-1.96 (m, 22H), 2.66 (d, J = 7.3 Hz, 2H) ppm; ^{13}C-NMR (100 MHz, $CDCl_3$): 18.63,

26,65, 28.94, 33.40, 33.93, 36.44, 41.53, 46.76, 108.16, 110.13 ppm; MS (EI, *m/z*): 310.36[M + H]$^+$.

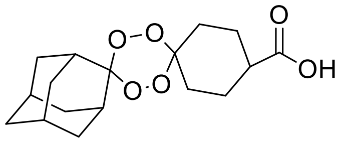

Dispiro[cyclohexane-1,3'-[1,2,4,5]tetraoxane-6',2''-tricyclo[3.3.1.1^{3,7}]decane]-4-carboxylic acid, LC153.[9]

To a solution of **LC138** (4 mmol) in methanol (15 mL) was added a solution of potassium hydroxide (20 mmol) in water (6 mL). The mixture was refluxed for 6 hours. Then the solution was allowed to cool to room temperature and concentrated under reduced pressure. The crude was taken up in water (50 ml) and washed with dichloromethane (30 ml). The aqueous layer was acidified to pH 1 with concentrated hydrochloric acid and then extracted with dichloromethane (3 x 40 ml). The combined organic phases were washed with brine (30 ml), dried over Na_2SO_4, filtered and concentrated under reduced pressure to give the pure compound as a white solid (80% yield). m.p. 179-182 ℃. ^{1}H-NMR (400 MHz, $CDCl_3$): δ 1.18-1.85 (m, 20H), 2.29-2.30 (m, 1H), 2.90 (brs, 1H), 3.17 (brs, 1H) ppm; ^{13}C-NMR (100 MHz, $CDCl_3$): 25.05, 26.06, 27.24, 32.97, 34.58, 35.04, 36.57, 36.96, 41.17, 106.95, 111.73, 181.13 ppm; MS (EI, *m/z*): 323.29 [M-H]$^-$.

General Procedure 4: Preparation of Adamantyl-1,2,4,5-tetraoxanes PA5 and LC163. To a solution of compound **LC140** (3.4 mmol) in anhydrous 1,2-dichloroethane (20 mL) was added amino compounds (3.74 mmol) and acetic acid (3.4 mmol). The mixture was allowed to stir at room temperature for 30 minutes followed by addition of sodium triacetoxyborohydride (8.5 mmol). After stirring at room temperature for 16 hours, the reaction mixture was washed with aqueous NaOH (5M; 2 x 10 mL) and dichloromethane (2 x 20 mL). The organic extract was dried over $MgSO_4$, filtered, and the solvent removed. The crude product was purified by column chromatography (silica gel, ethyl acetate/n-hexane 3/7) to give product.

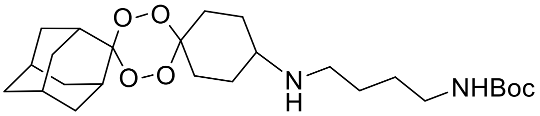

4-[4-(*Tert*-butoxycarbonylamino)butylamino]dispiro[cyclohexane-1,3'-[1,2,4,5]tetraoxane-6',2''-tricyclo[3.3.1.1^{3,7}]decane], PA5.

Prepared according to general procedure 4 to give **PA5** as yellow oil (32% yield). ^{1}H-NMR (400 MHz, $CDCl_3$): δ 0.93 (m, 3H), 1.34 (d, *J* = 6.9 Hz, 3H), 1.47 (s, 9H), 1.49-1.76 (m, 12H), 2.06 (m, 8H), 2.86 (t, *J* = 7.0 Hz, 3H), 3.15 (s, 2H) ppm; ^{13}C-NMR (100 MHz, $CDCl_3$): 22.53, 23.42, 26.55, 27.37, 28.11, 30.01, 32.02, 34.31, 36.05, 38.21, 47.61, 60.53, 79.49, 108.90, 111.01, 156.14 ppm; MS (MALDI-TOF, *m/z*): 467,32 $[M+H]^+$.

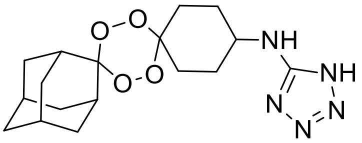

***N-1H*-1,2,3,4-Tetraazol-5-yl-dispiro[cyclohexane-1,3'-[1,2,4,5]tetraoxane-6',2''-tricyclo[3.3.1.1^{3,7}]decan]-4-ylamine, LC163.**[6]

Prepared according to general procedure 4 to give **LC163** as a white solid (95% yield); m.p. 142-144 ℃. ^{1}H-NMR (400 MHz, $CDCl_3$): δ 1.15-1.22 (m, 2H), 1.60-1.70 (m, 10H), 1.80-2.05 (m, 10H), 2.6 (m, 1H) ppm; ^{13}C-NMR (100 MHz, $CDCl_3$): 20.05, 26.06, 27.42, 29.67, 32.78, 33.64, 59.57, 106.44, 111.91 ppm; MS (MALDI-TOF, *m/z*): 363.43 $[M]^+$.

General Procedure 5: Preparation of Adamantyl-1,2,4,5-tetraoxanes PA6 and LC167. EDC.HCl (1.5eq), HOBt (1.5eq) and NMM (2.1eq) were added to **LC153** (1eq) in DCM (15ml) at 0 ℃. The solution was stirred at room temperature for 3hrs under N_2 before different amines (1.5eq) were added. After stirring at room temperature overnight, water (50ml) was added and the product extracted with Et_2O (3 x 30ml). The combined organic extracts were dried over Na_2SO_4, filtered and concentrated under reduced pressure and purified by flash column chromatography to afford the products.

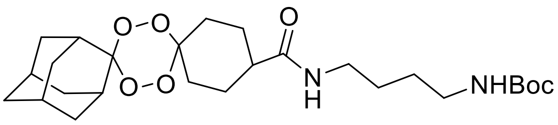

***N*-[4-(*Tert*-butoxycarbonylamino)butyl]-dispiro[cyclohexane-1,3'-[1,2,4,5]tetraoxane-6',2''-tricyclo[3.3.1.1^{3,7}]decane]-4-carboxamide, PA6.**

Prepared according to general procedure 5 to give **PA6** as a white solid (54% yield). m.p. 110-112 ℃. ^{1}H-NMR (400 MHz, CDCl$_3$): δ 1.24 (m, 3H), 1.48-1.49 (m, 9H), 1.61-1.86 (m, 3H), 1.94-2.09 (m, 8H), 2.41 (d, *J* = 6.5 Hz, 1H), 3.11 (t, *J* = 7.1 Hz, 2H), 3.23 (m, 2H) ppm; ^{13}C-NMR (100 MHz, CDCl$_3$): 17.92, 23.75, 27.35, 27.77, 28.41, 31.93, 33.55, 35.42, 36.16, 39.67, 108.81, 110.92, 156.40, 178.20 ppm; MS (MALDI-TOF, *m/z*): 495,29 [M + H]$^+$.

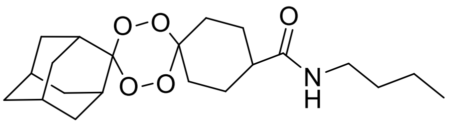

***N*-Butyl-dispiro[cyclohexane-1,3'-[1,2,4,5]tetraoxane-6',2''-tricyclo[3.3.1.1^{3,7}]decane]-4-carboxamide, LC167.**

Prepared according to general procedure 5 to give **LC167** as light yellow oil. (20% yield). ^{1}H-NMR (400 MHz, CDCl$_3$): δ 1.18-1.22 (m, 3H), 1.66 (m, 4H), 1.72-1.77 (m, 6H), 1.84-1.98 (m, 10H), 1.98-2.10 (m, 6H), 2.44 (m, 2H), 2.99 (t, *J* = 7.2 Hz, 2H) ppm; ^{13}C-NMR (100 MHz, CDCl$_3$): 14.06, 17.92, 19.87, 23.75, 27.77, 28.41, 31.93, 33.55, 36.16, 39.67, 25.42, 27.14, 33.23, 37.04, 107.81, 109.92 ppm; MS (EI, *m/z*): 380.25 [M+H]$^+$.

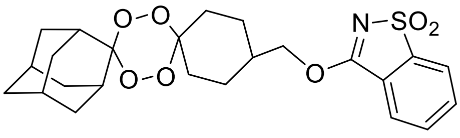

3-{(Dispiro[cyclohexane-1,3'-[1,2,4,5]tetraoxane-6',2''-tricyclo[3.3.1.1^{3,7}]decan]-4-yl)methoxy}-*1H*-1λ^6,2-benzisothiazole-1,1-dione, LC177.

Compound **LC146** (4.08 mmol) was added to solution of compound **LC60** (3.4 mmol) in dry Toluene (30 mL). The solution was stirred at 45 ℃ for 15 minutes followed by triethylamine (6.8 mmol) until all of the starting material had disappeared. The precipitate of triethylamine hydrochloride was filtered off and the filtrate was evaporated to give a yellow crystalline solid, which was recrystallized from ethanol (21% yield); m.p. 141-142 ℃. ^{1}H-NMR (400 MHz, CDCl$_3$): δ 1.32-1.35 (m, 2H), 1.62-1.99 (m, 21H), 4.16 (d, *J* = 7.0 Hz, 2H), 7.64 (m,

1H), 7.68 (m, 2H), 7.82 (d, *J* = 7.2 Hz, 1H) ppm; ^{13}C-NMR (100 MHz, $CDCl_3$): 17.6, 26.87, 29.24, 32.36, 35.39, 37.95, 39.13, 66.39, 108.22, 111.62, 123.30, 127.00, 133.43, 134.11, 143.61, 169.22 ppm; MS (EI, *m/z*): 476.16 $[M+H]^+$.

General Procedure 6: Preparation of Adamantyl-1,2,4,5-tetraoxanes LC179 and LC180. To a solution of **LC146** (1.83 mmol) in THF (10 mL) was added mesyl chloride (2.0 mmol) and triethylamine (3.65 mmol). The solution was stirred at room temperature for 3 hours. Then a solution of **LC126II or LC126II** (2.75 mmol) in THF (10 mL) was added dropwise to the stirred suspension over 30 minutes. The mixture was stirred at 65 ℃ for 24 hours. Excess solvent was then removed. Recrystallization from ethanol gave the desired compound.

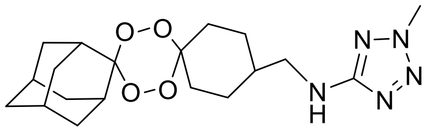

{(Dispiro[cyclohexane-1,3'-[1,2,4,5]tetraoxane-6',2''-tricyclo[3.3.1.1^{3,7}]decan]-4-yl)methyl}(2-methyl-*2H*-1,2,3,4-tetraazol-5-yl)amine, <u>LC179</u>.

Prepared according to general procedure 6 to give **LC179** as a white solid (25% yield). m.p. 134-136 ℃. ^{1}H-NMR (400 MHz, $CDCl_3$): δ 1.06-1.09 (m, 2H), 1.17-1.24 (m, 10H), 1.62-1.64 (m, 3H), 1.73-1.85 (m, 6H), 2.04 (d, *J* = 7.1 Hz, 2H), 2.95 (d, *J* = 7.4 Hz, 2H), 3.85 (d, *J* = 6.5 Hz, 3H) ppm; ^{13}C-NMR (100 MHz, $CDCl_3$): 18.93, 26.25, 28.44, 33.00, 33.70, 36.53, 38.56, 41.53, 54.96, 108.16, 110.13 ppm; MS (EI, *m/z*): 391.31 $[M]^+$.

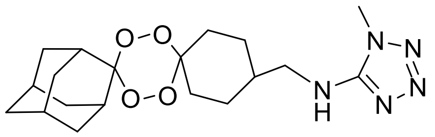

{(Dispiro[cyclohexane-1,3'-[1,2,4,5]tetraoxane-6',2''-tricyclo[3.3.1.1^{3,7}]decan]-4-yl)methyl}(1-methyl-*1H*-1,2,3,4-tetraazol-5-yl)amine, <u>LC180</u>.

Prepared according to general procedure 6 to give **LC180** as yellow oil (32% yield). ^{1}H-NMR (400 MHz, $CDCl_3$): 1.07-1.09 (m, 2H), 1.17-1.23 (m, 10H), 1.62-1.64 (m, 3H), 1.73-1.87 (m, 6H), 2.06 (d, *J* = 7.1 Hz, 2H), 3.15 (d, *J* = 7.4 Hz, 2H), 4.15 (d, *J* = 6.5 Hz, 3H) ppm; ^{13}C-NMR (100 MHz, $CDCl_3$): 19.73, 27.23, 29.46, 34.02, 34.70, 37.55, 39.55, 42.54, 55.96, 108.05, 110.01 ppm; MS (EI, *m/z*): 391.29 $[M]^+$.

S.2. Spectra of the compounds

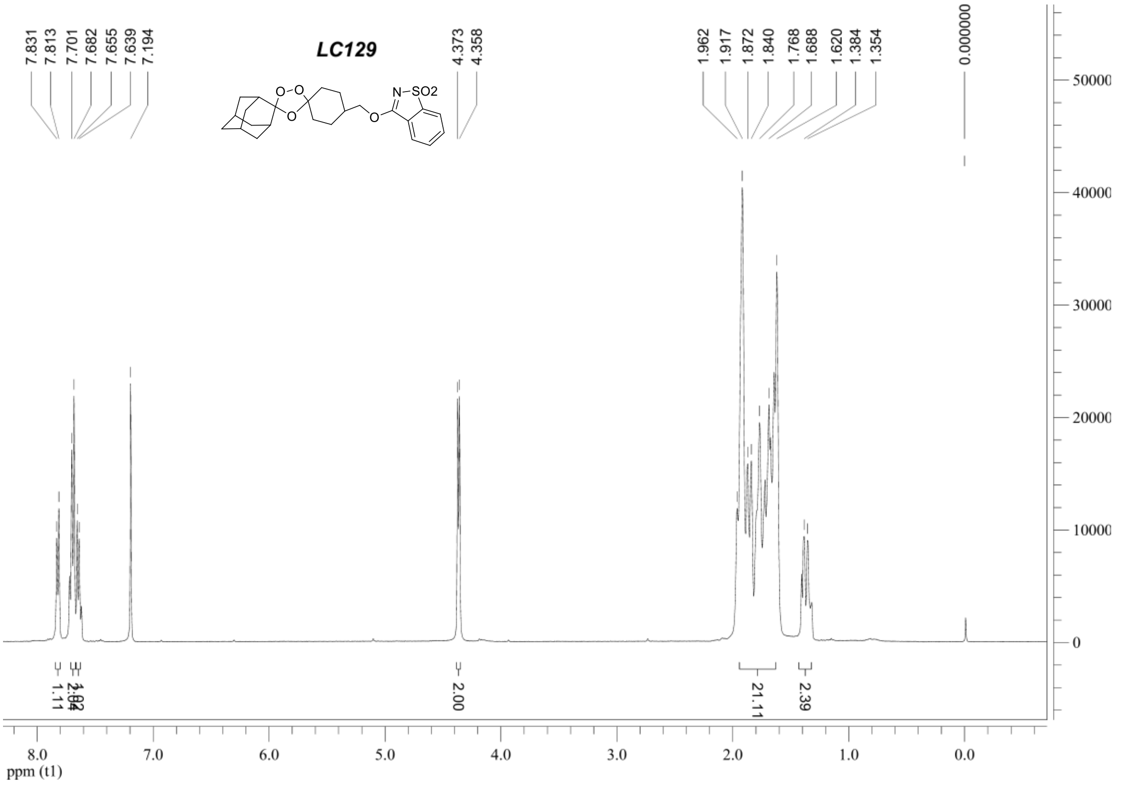

Figure 1. ^{1}H NMR spectrum of compound LC129.

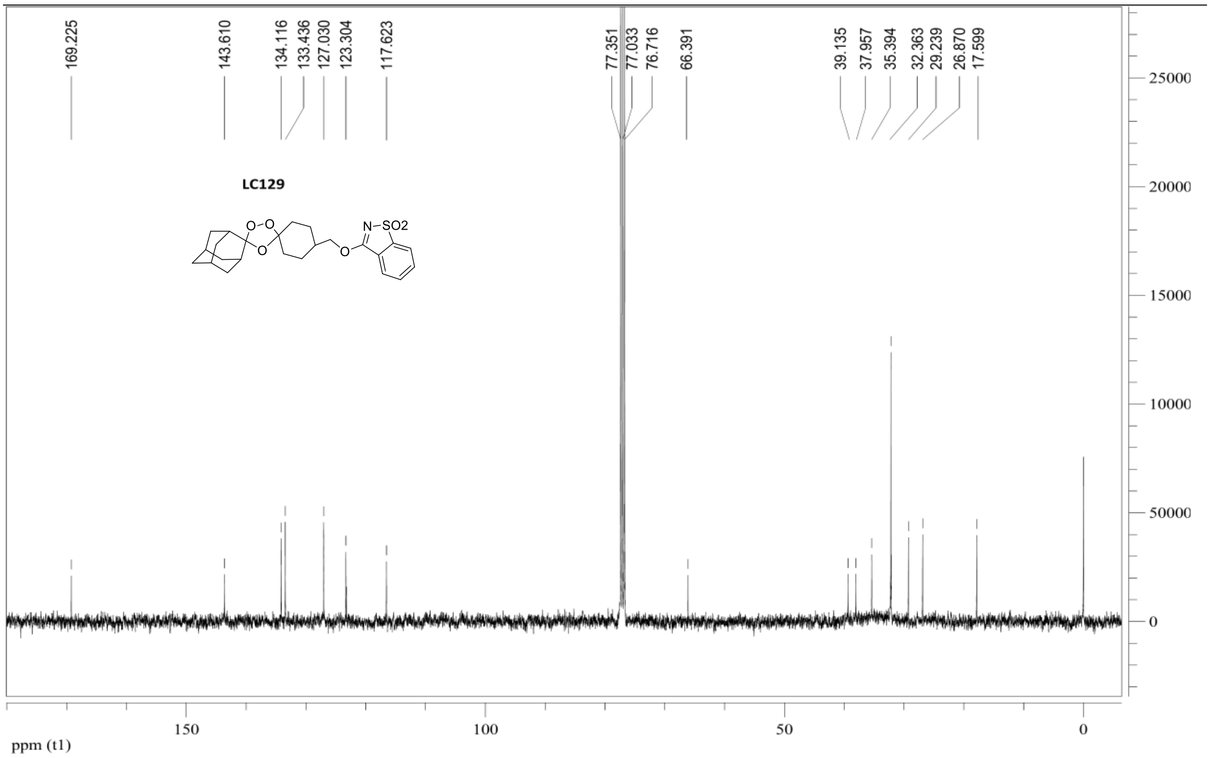

Figure 2. ^{13}C NMR spectrum of compound LC129.

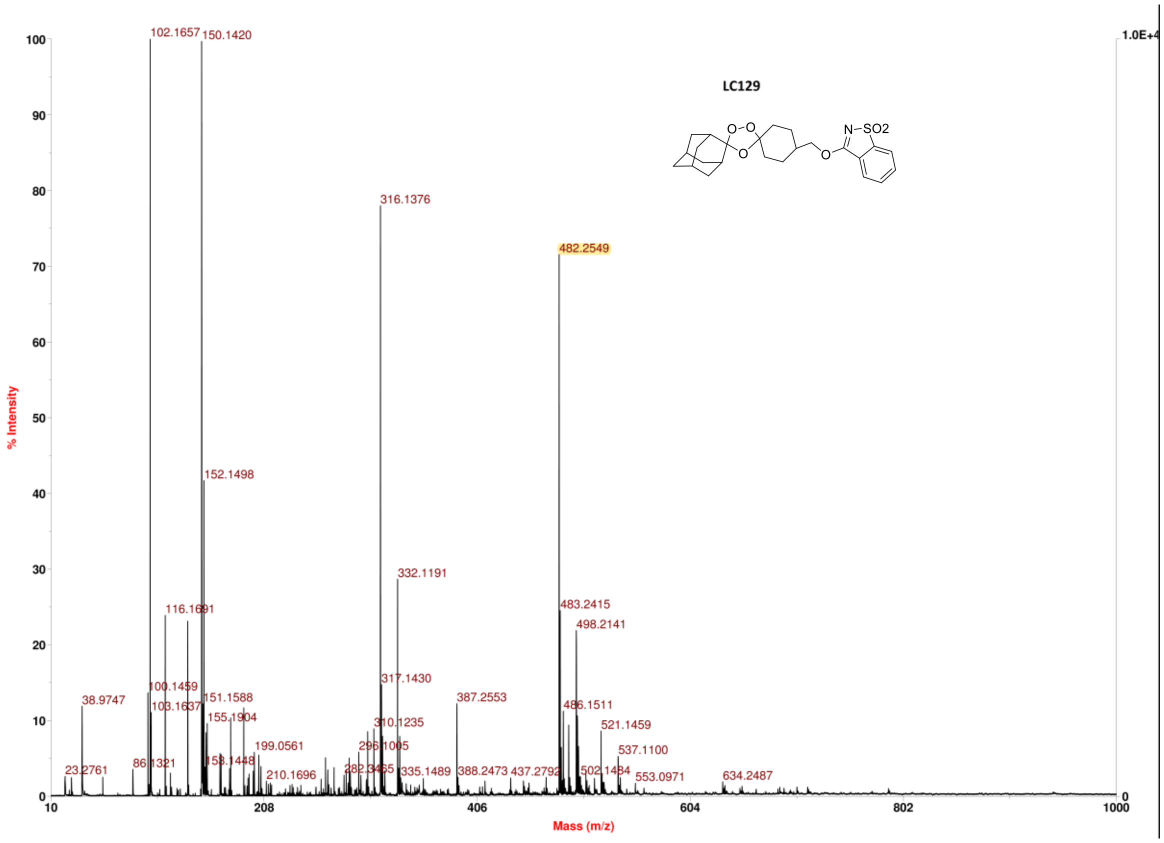

Figure 3. Mass spectrum of compound LC129.

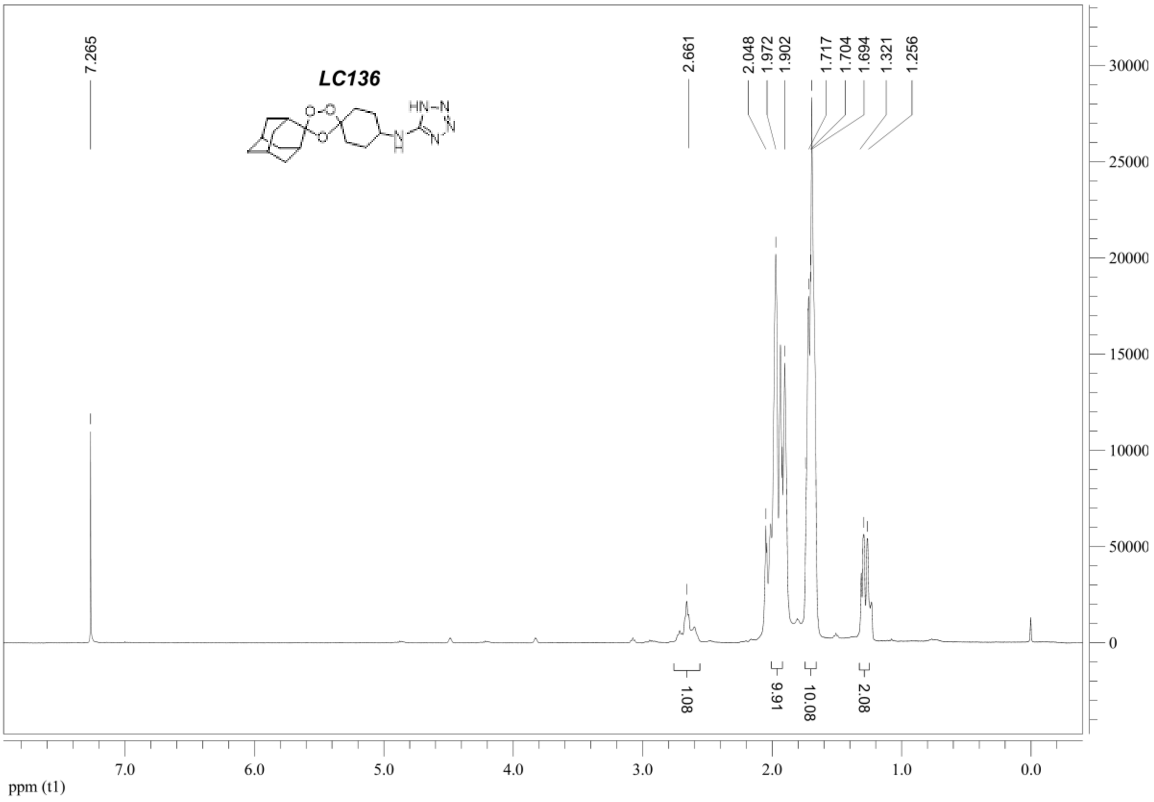

Figure 4. ^{1}H NMR spectrum of compound LC136.

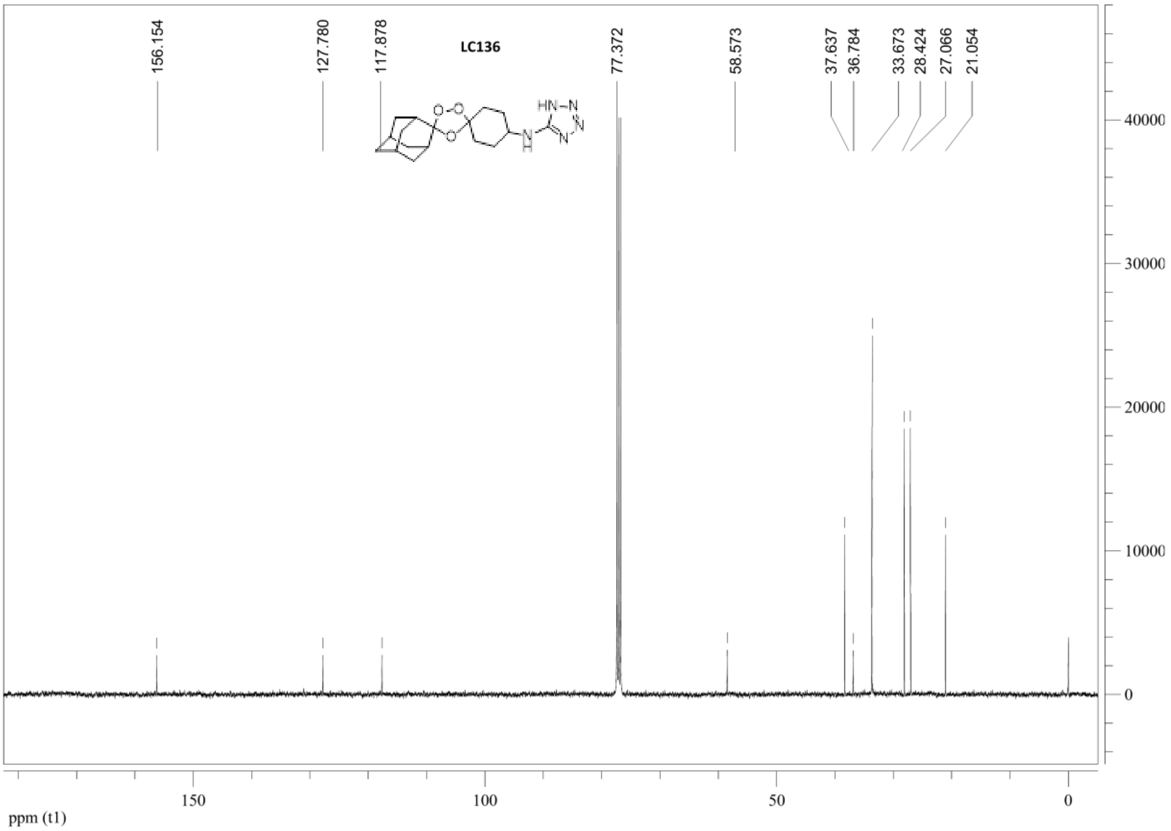

Figure 5. ^{13}C NMR spectrum of compound LC136.

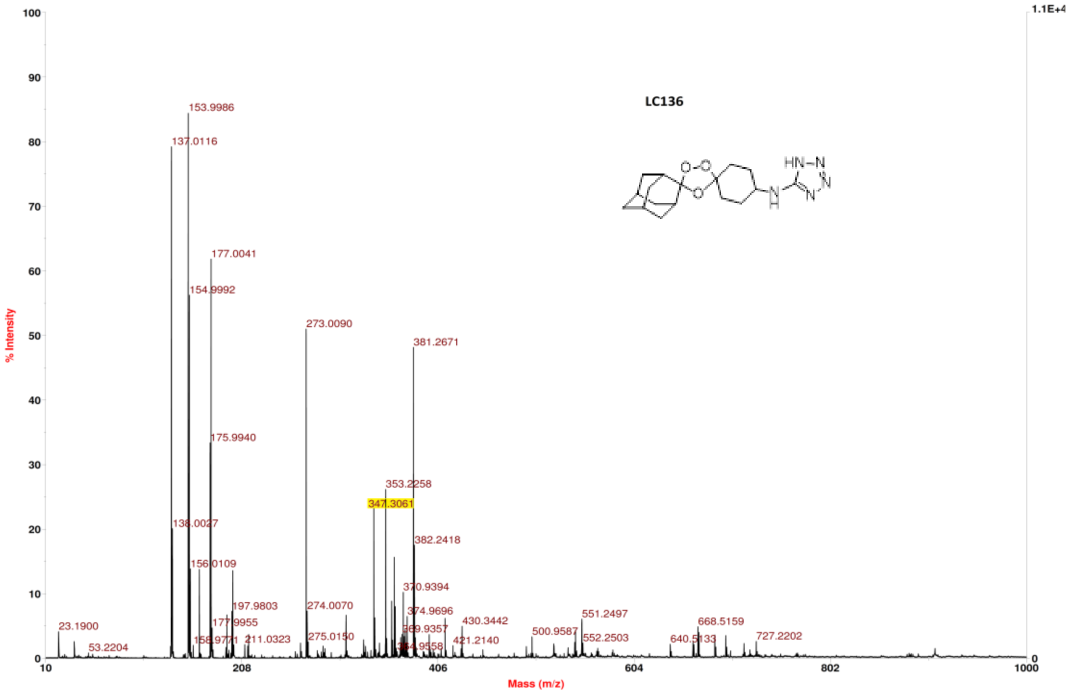

Figure 6. Mass spectrum of compound LC136.

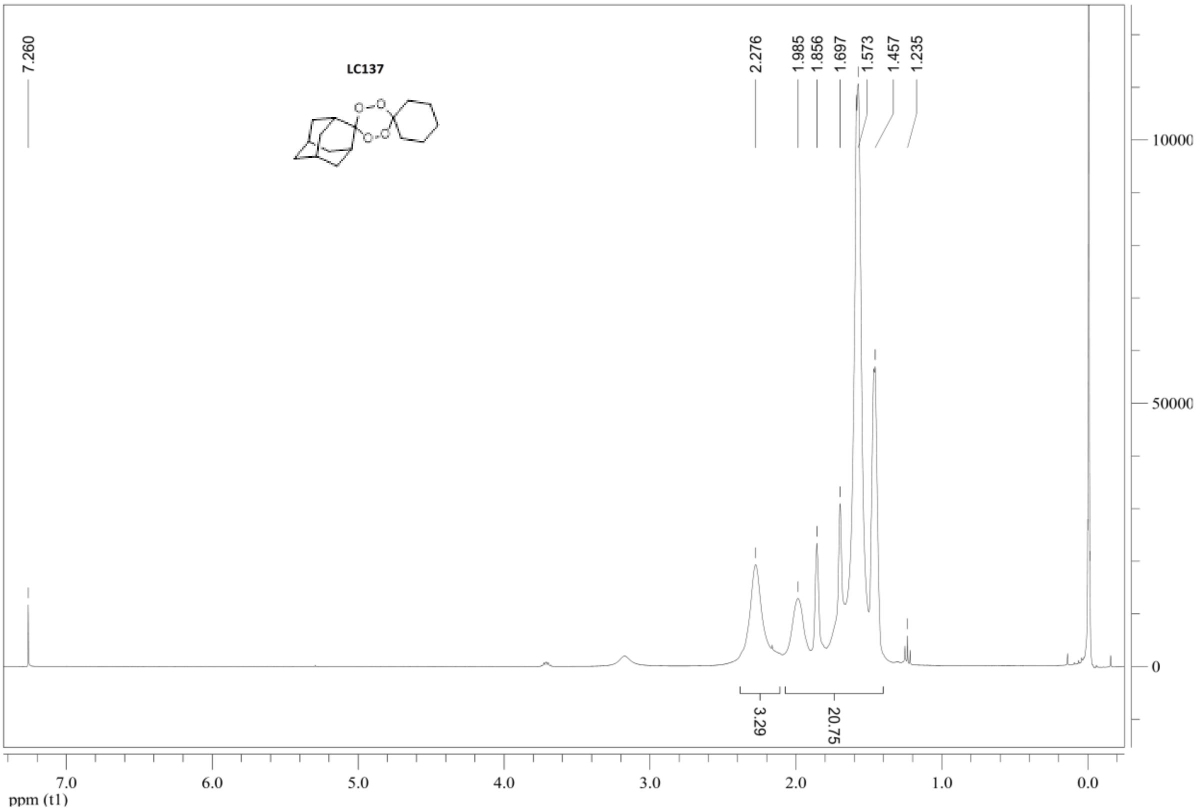

Figure 7. ^{1}H NMR spectrum of compound LC137.

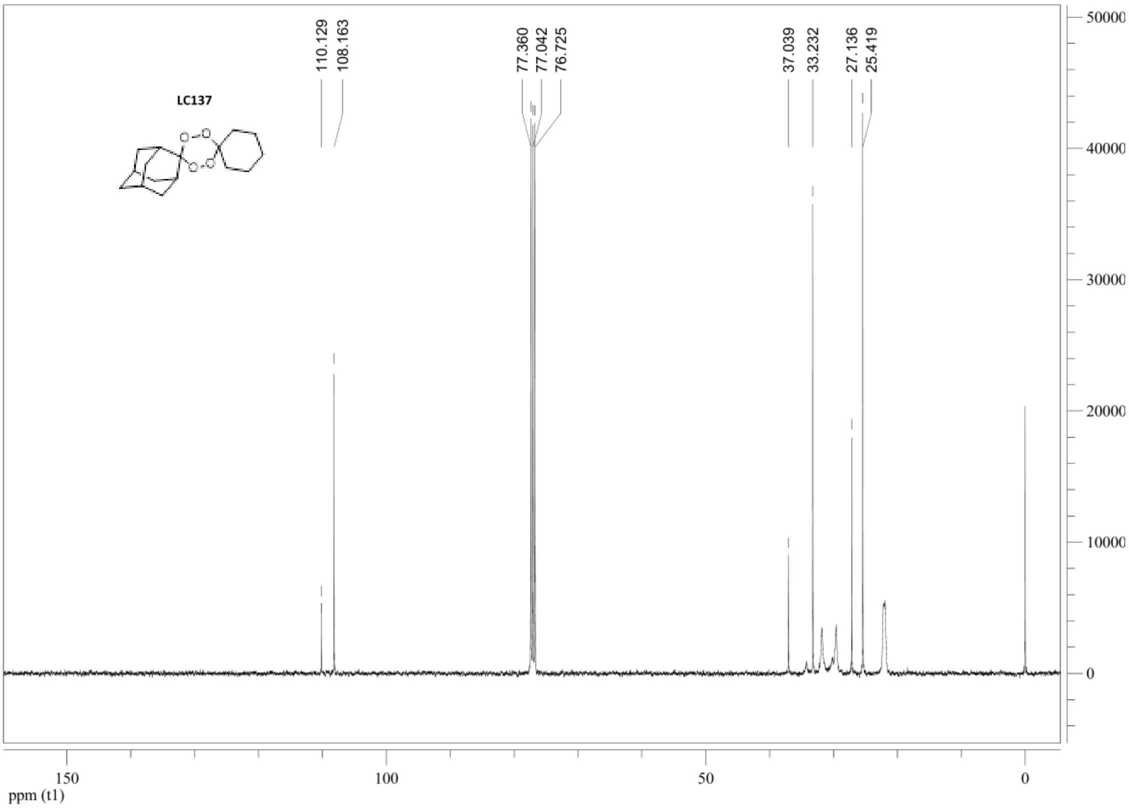

Figure 8. ^{13}C NMR spectrum of compound LC137.

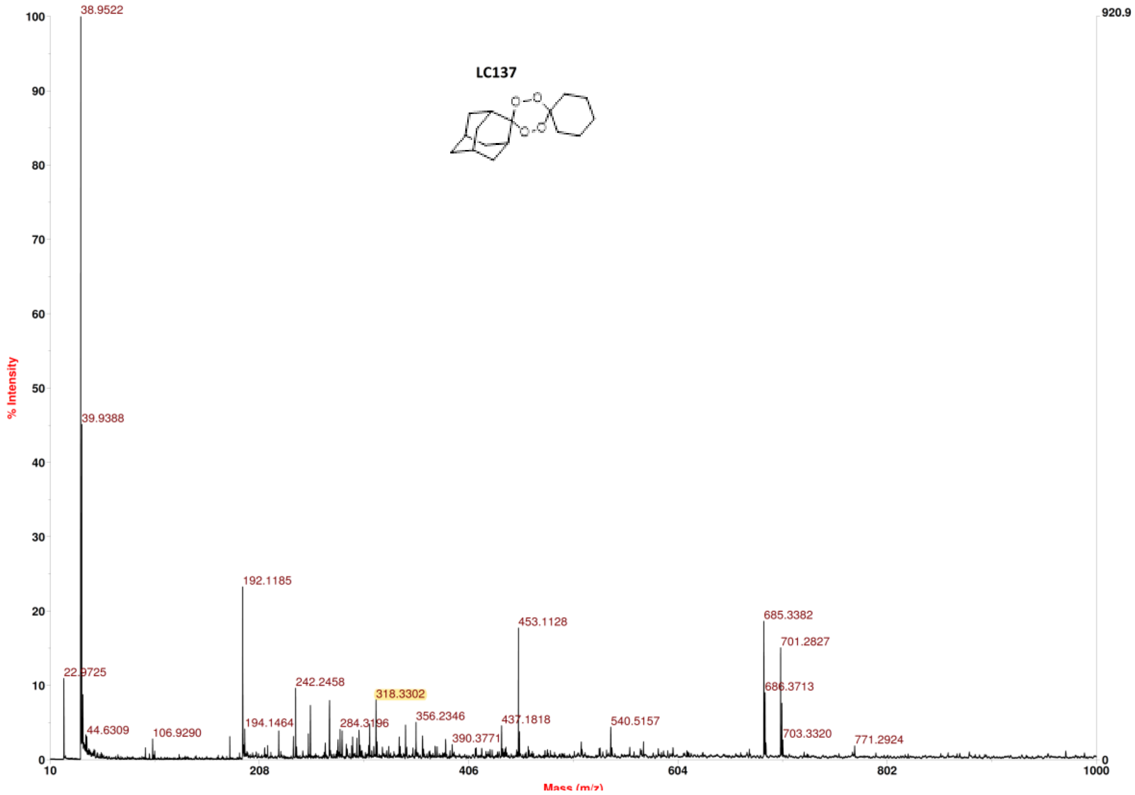

Figure 9. Mass spectrum of compound LC137.

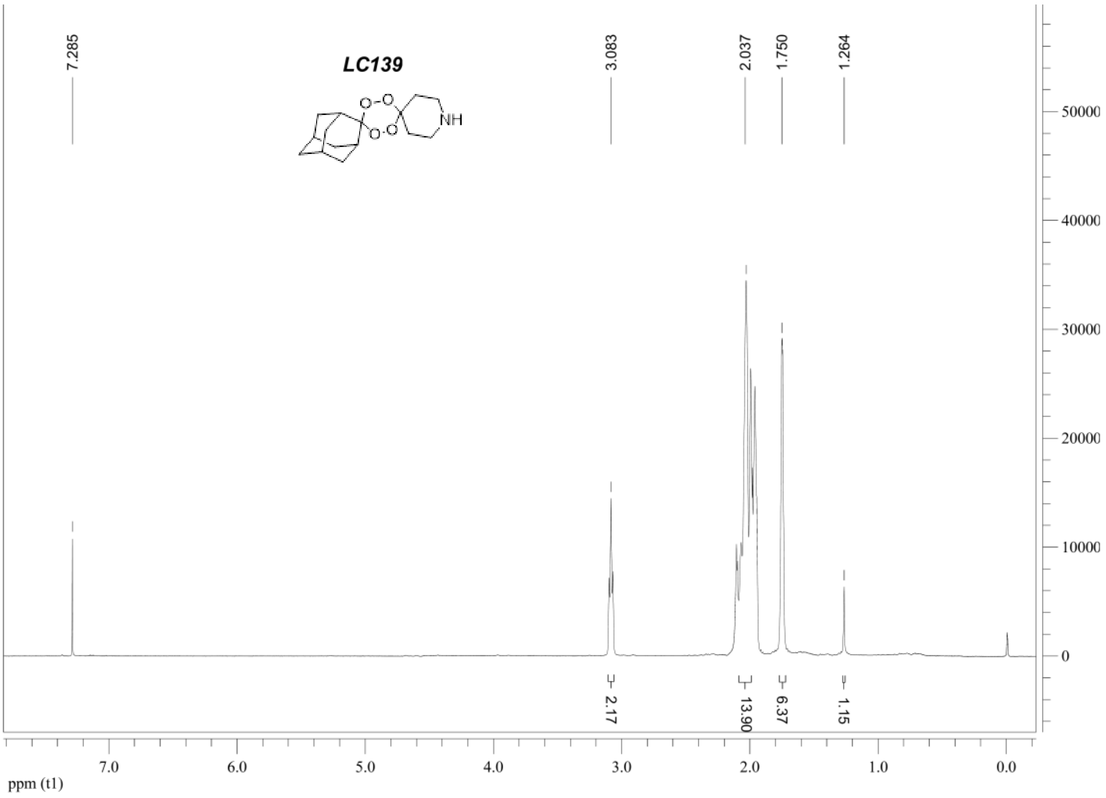

Figure 10. ^{1}H NMR spectrum of compound LC139.

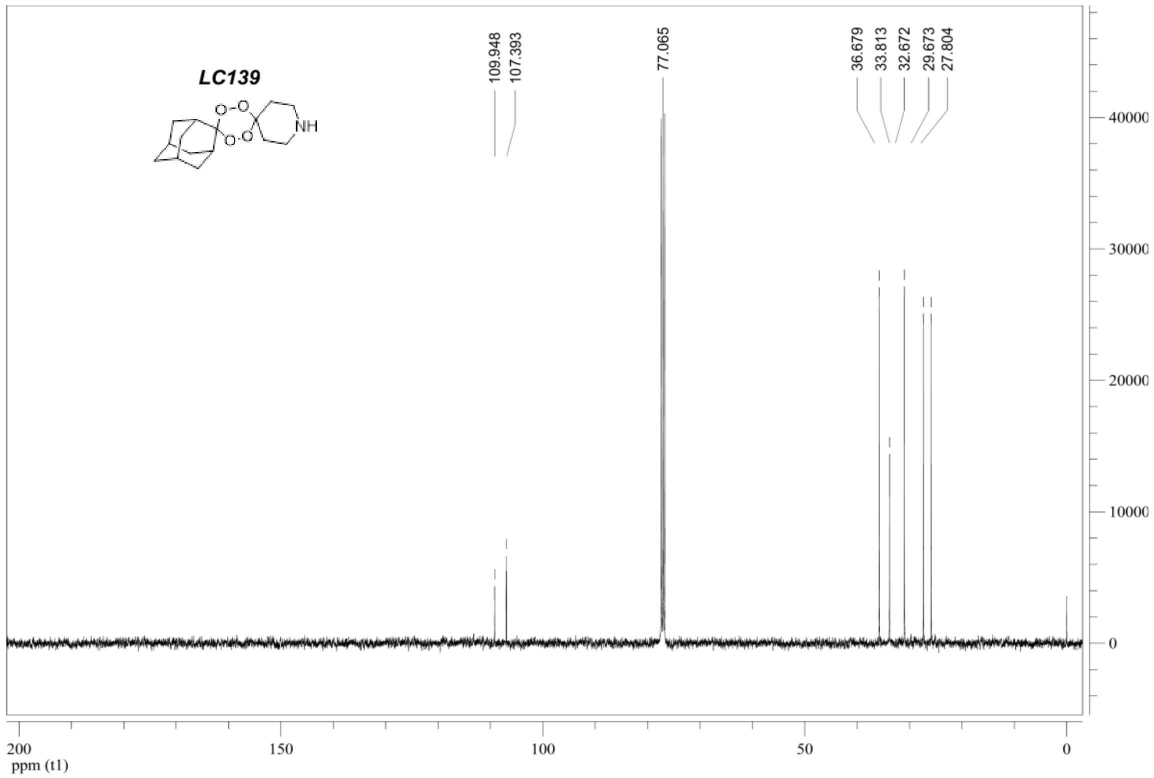

Figure 11. ^{13}C NMR spectrum of compound LC139.

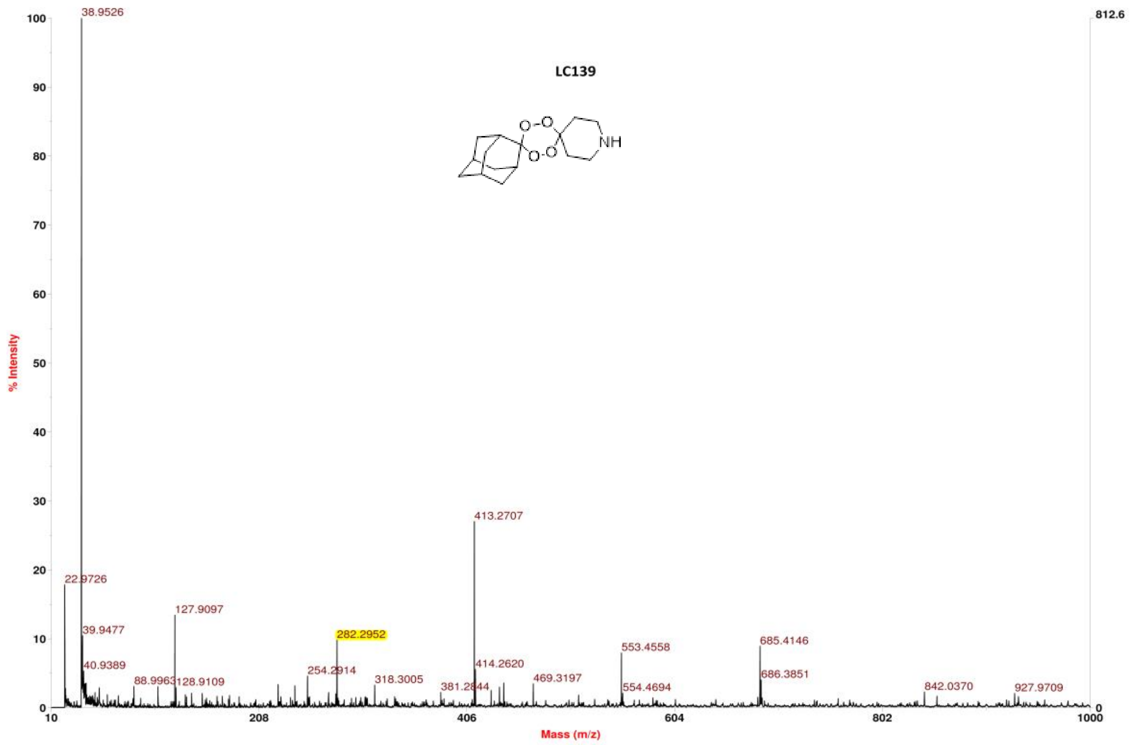

Figure 12. Mass spectrum of compound LC139.

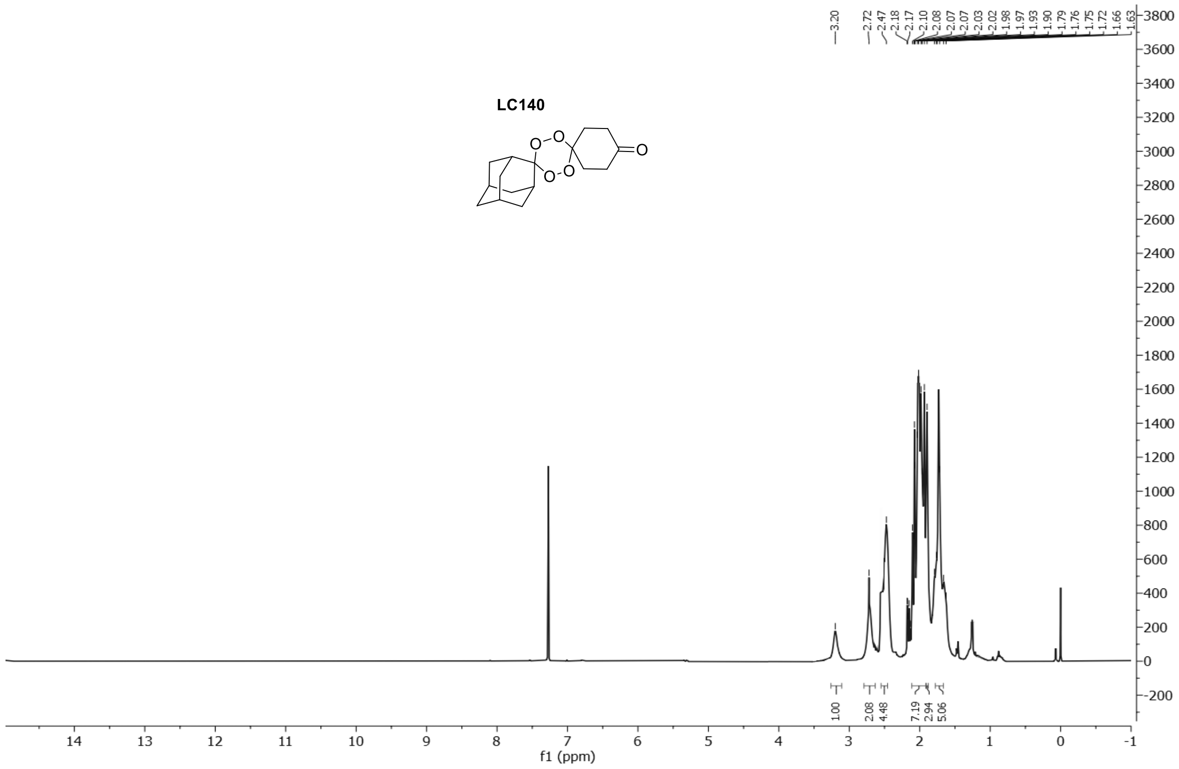

Figure 13. ^{1}H NMR spectrum of compound LC140

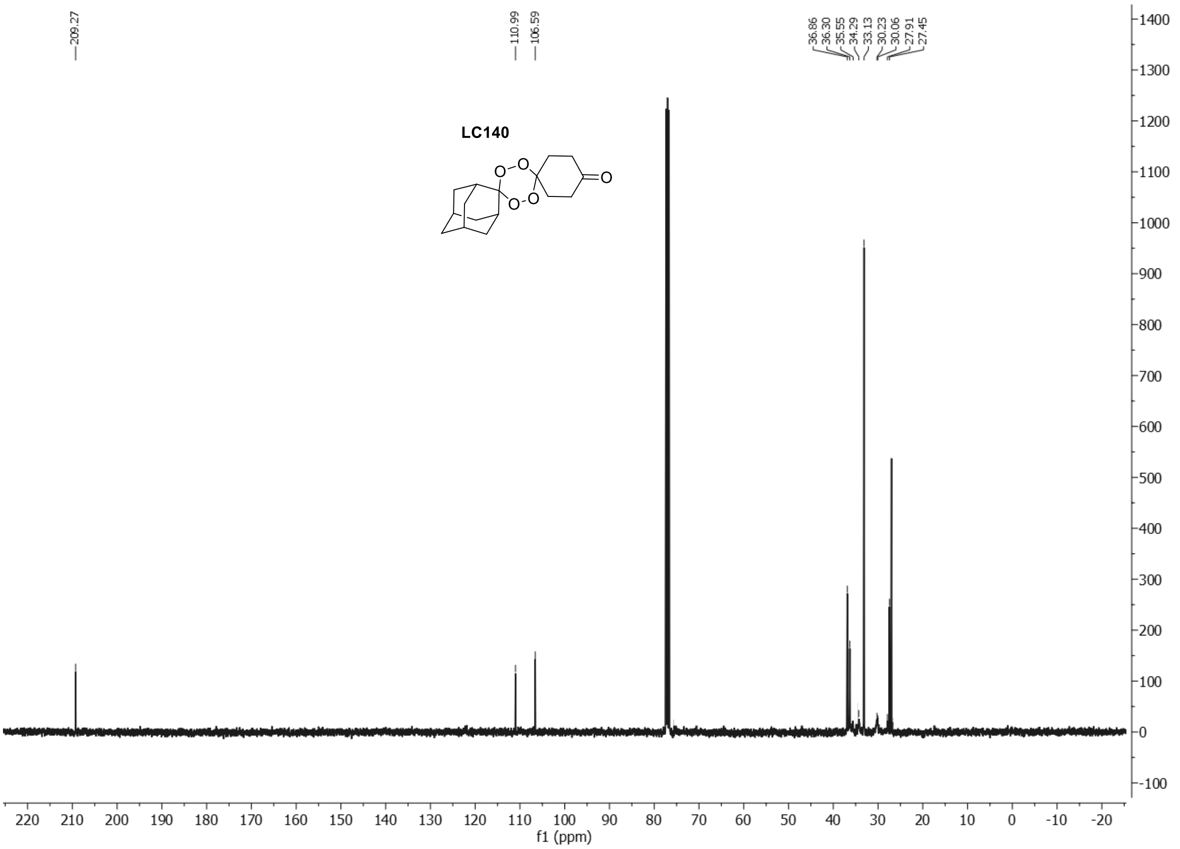

Figure 14. ^{13}C NMR spectrum of compound LC140

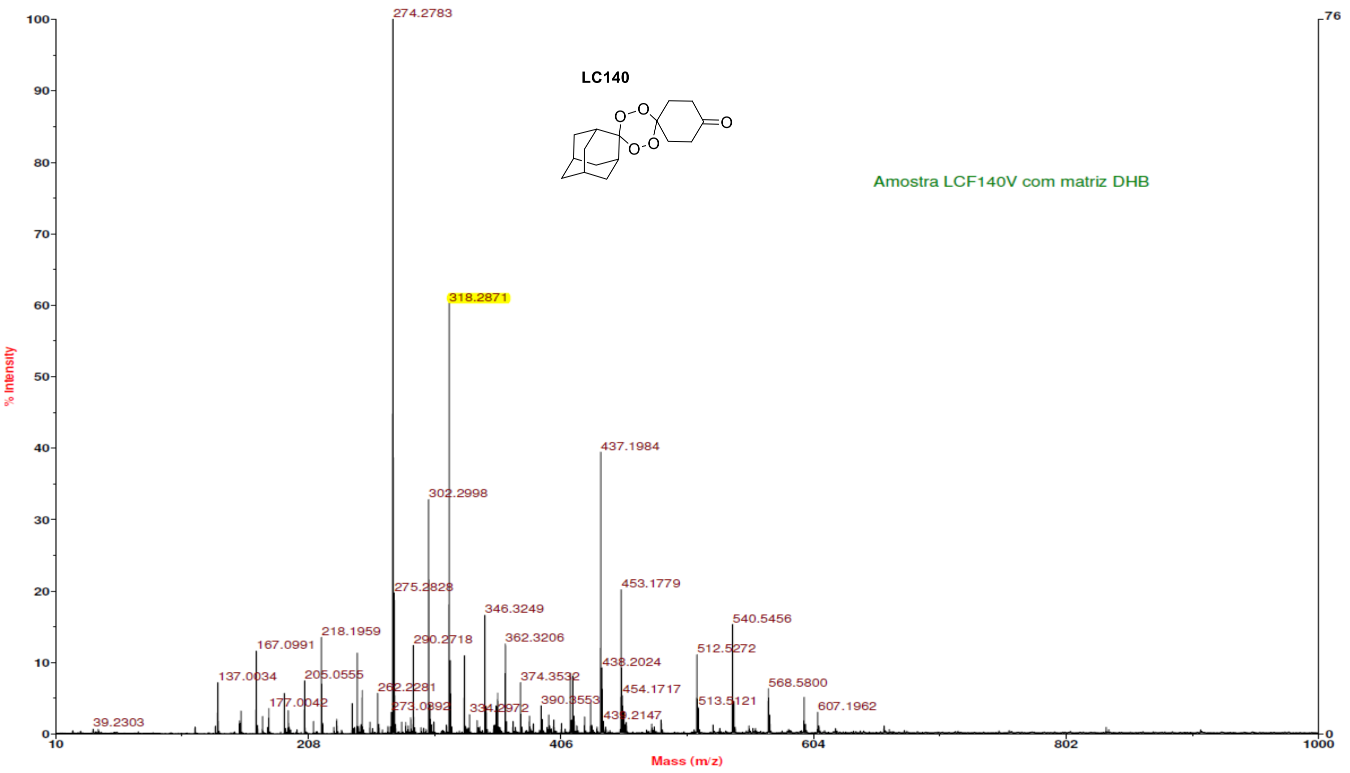

Figure 15. Mass spectrum of compound LC140.

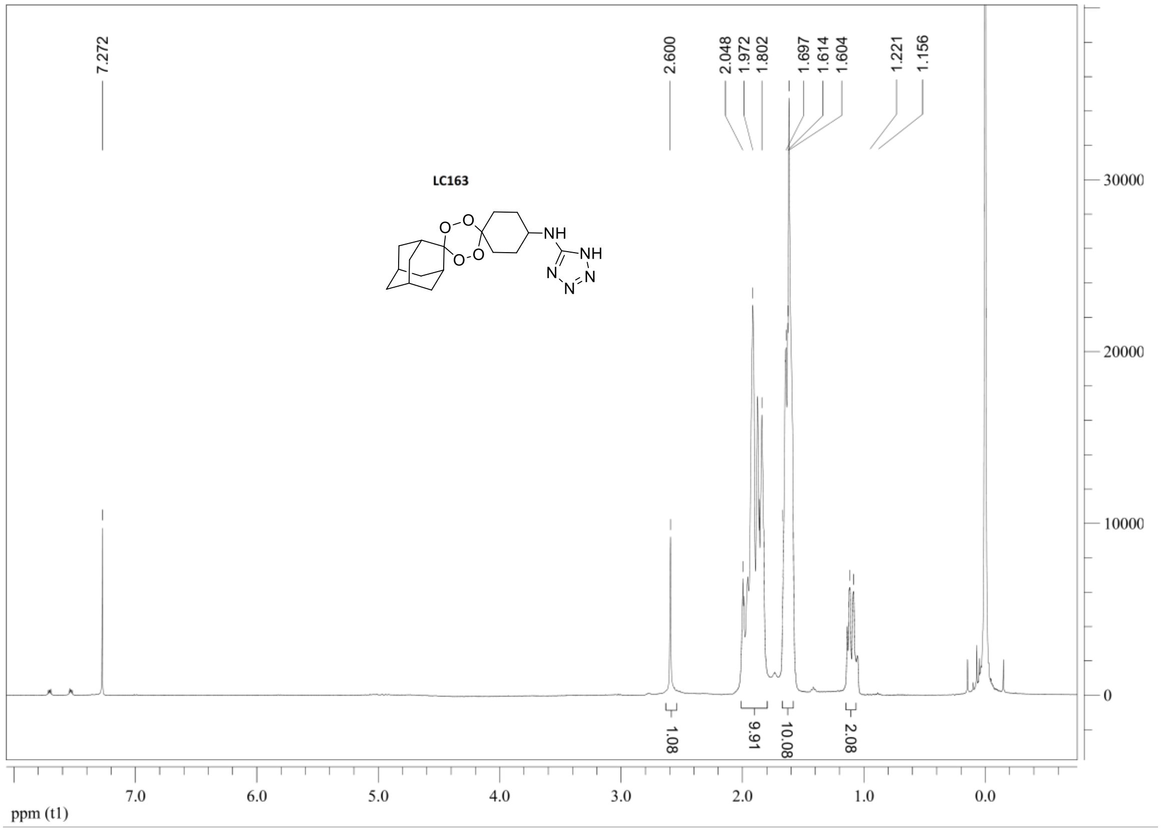

Figure 16. ^{1}H NMR spectrum of compound LC163.

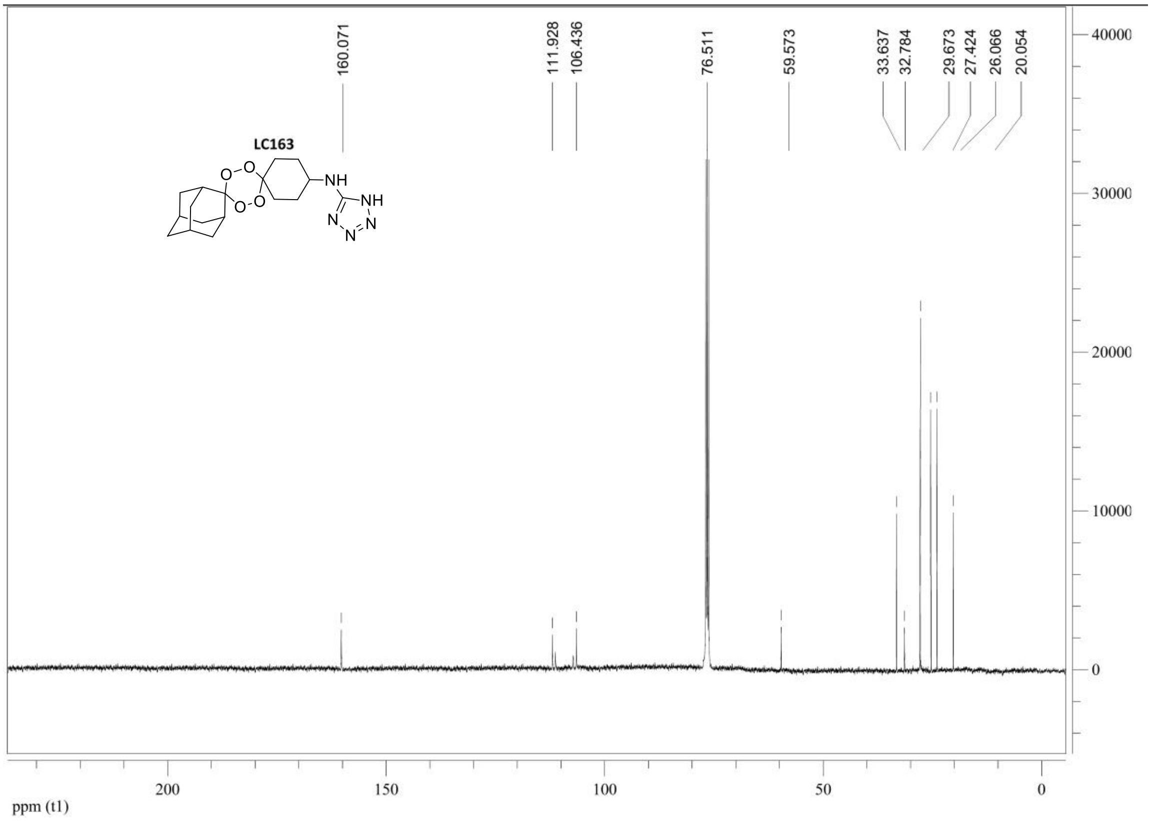

Figure 17. ^{13}C NMR spectrum of compound LC163.

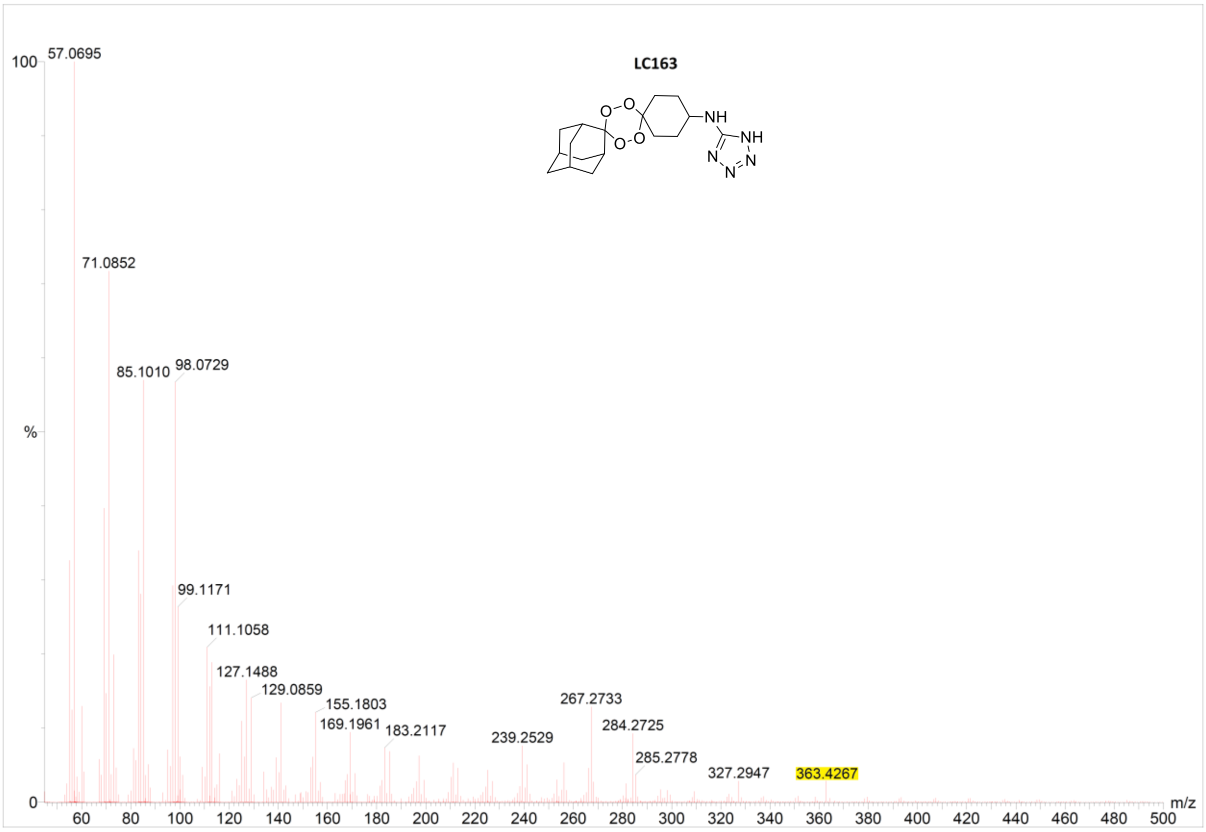

Figure 18. Mass spectrum of compound LC163.

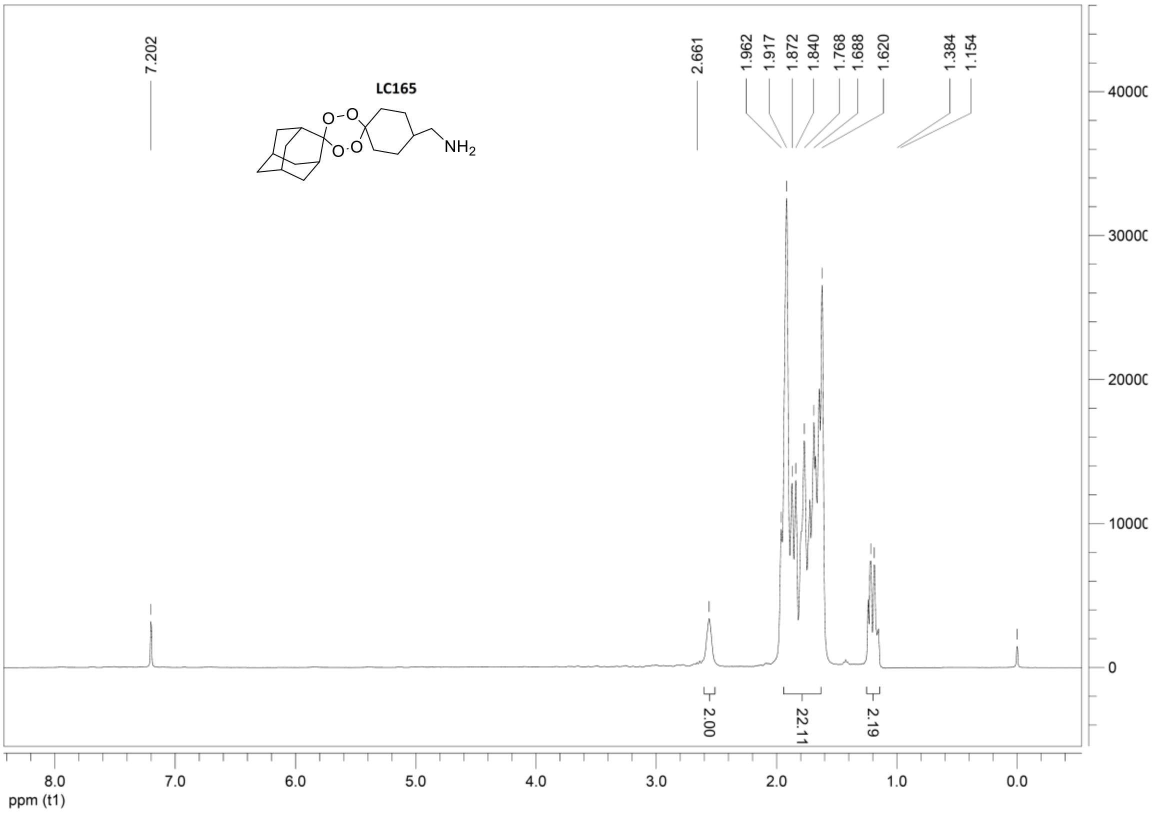

Figure 19. ^{1}H NMR spectrum of compound LC165.

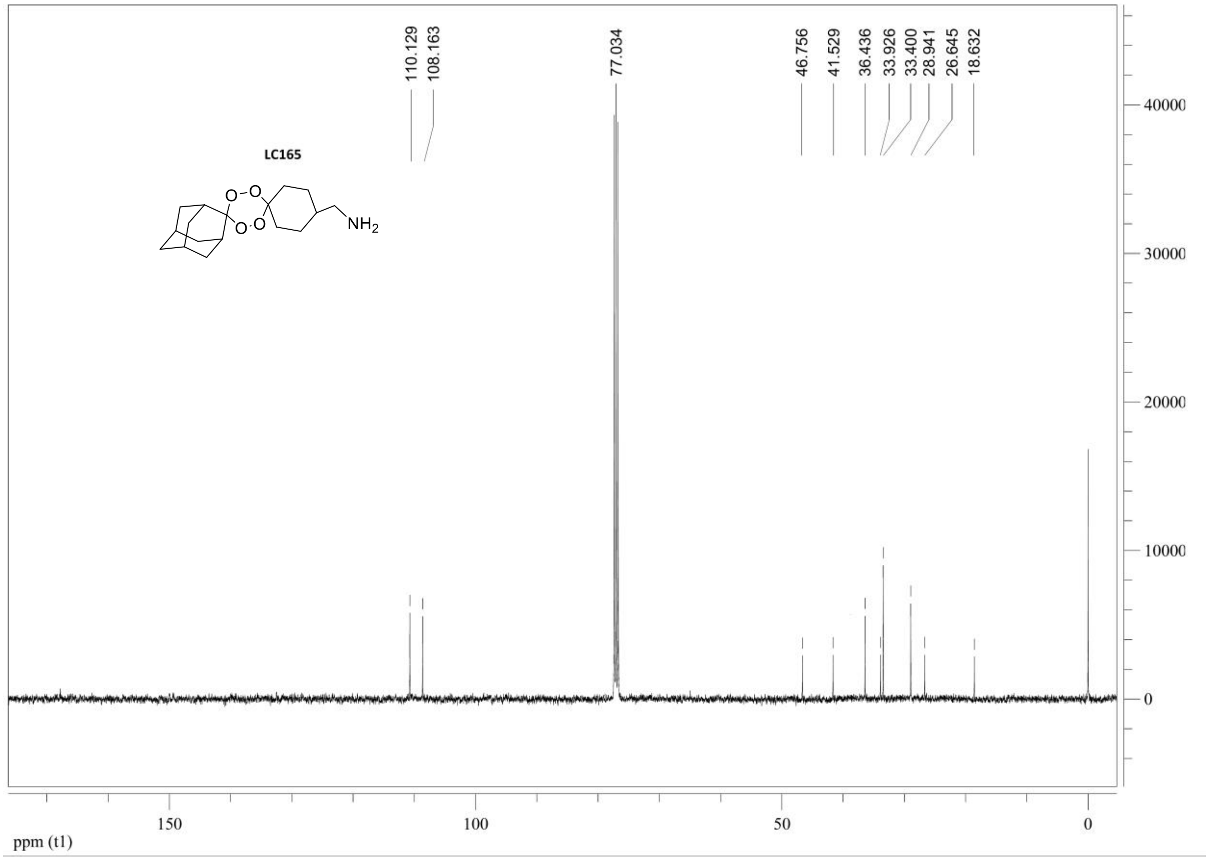

Figure 20. ^{13}C NMR spectrum of compound LC165.

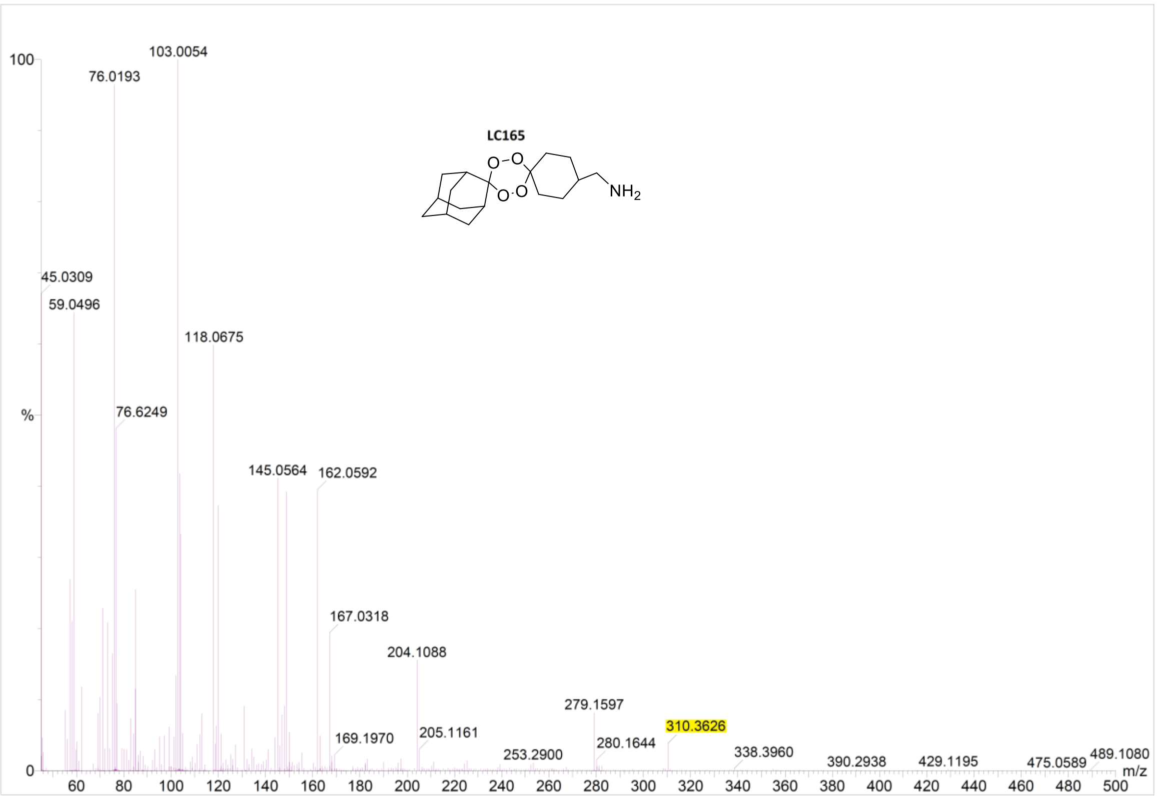

Figure 21. Mass spectrum of compound LC165.

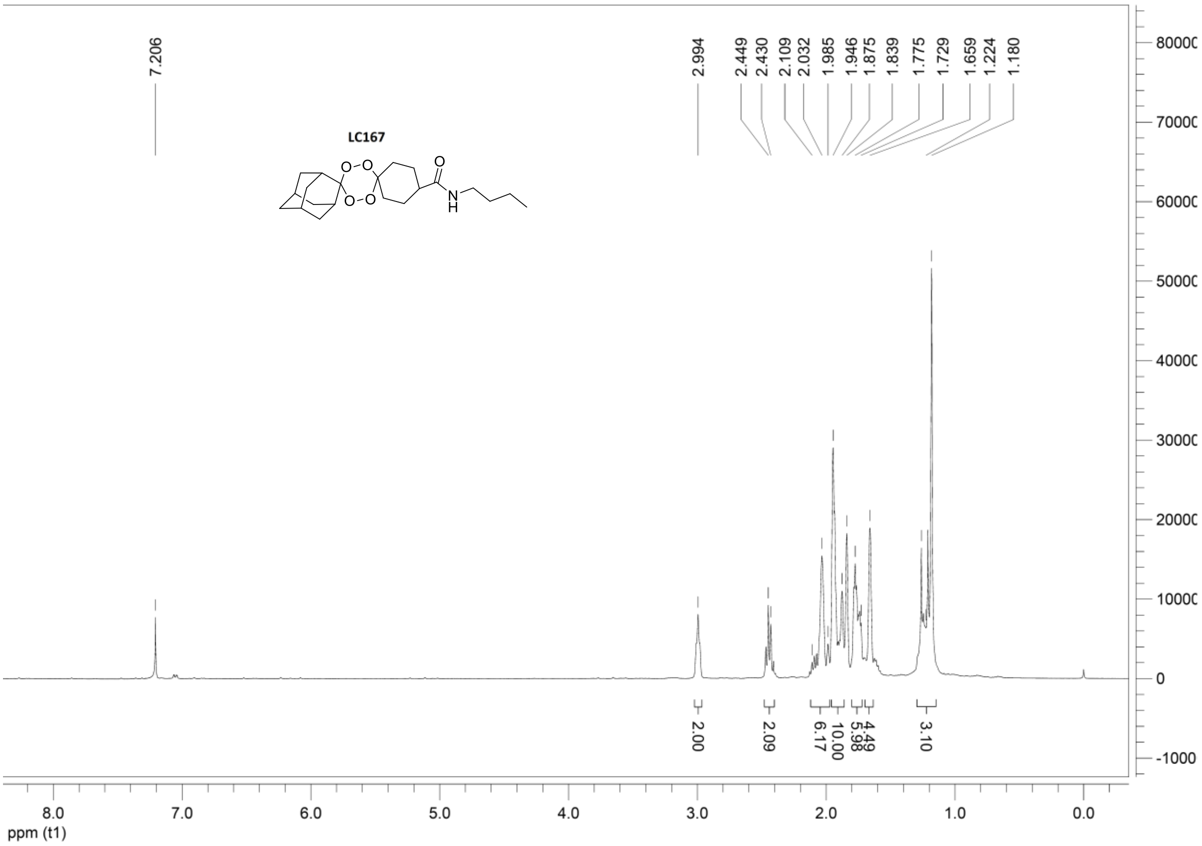

Figure 22. ^{1}H NMR spectrum of compound LC167.

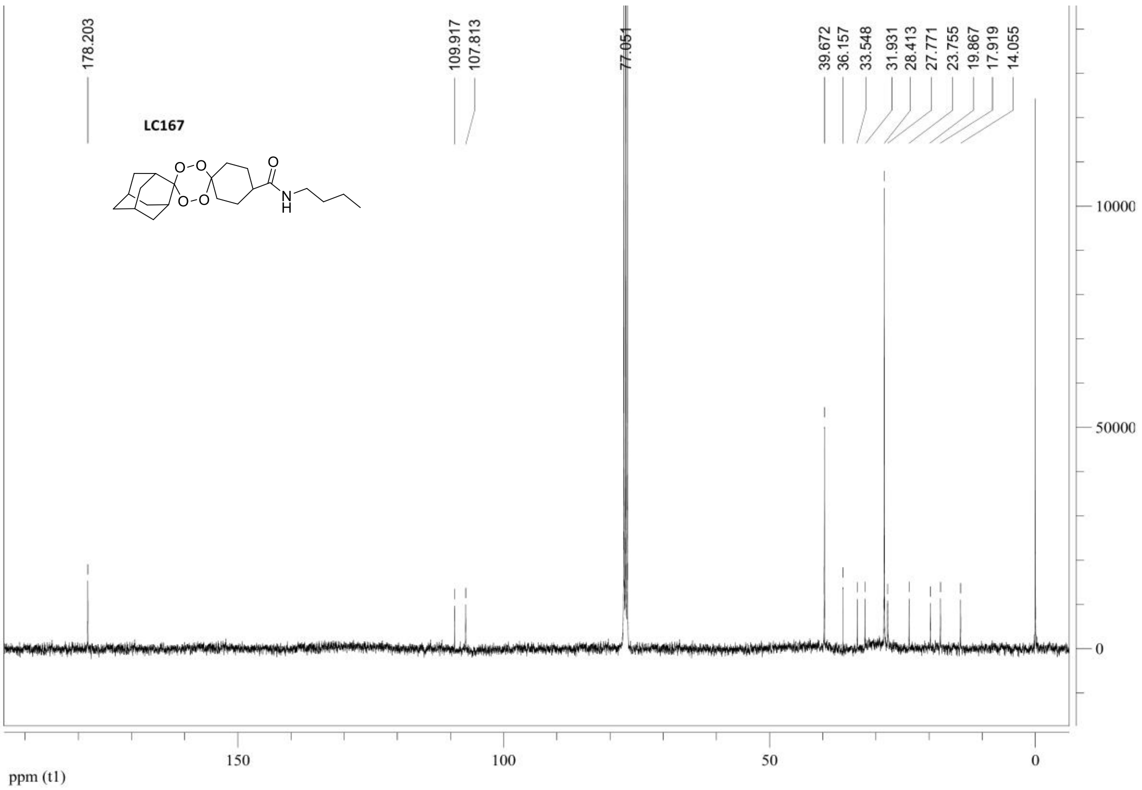

Figure 23. ^{13}C NMR spectrum of compound LC167.

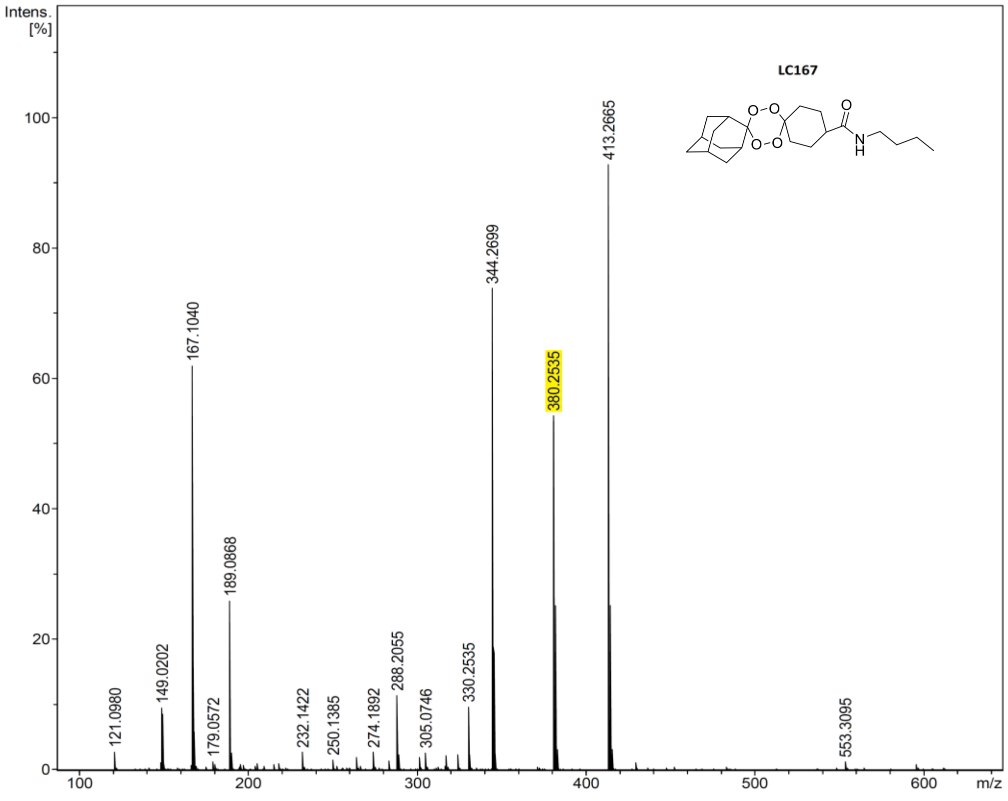

Figure 24. Mass spectrum of compound LC167.

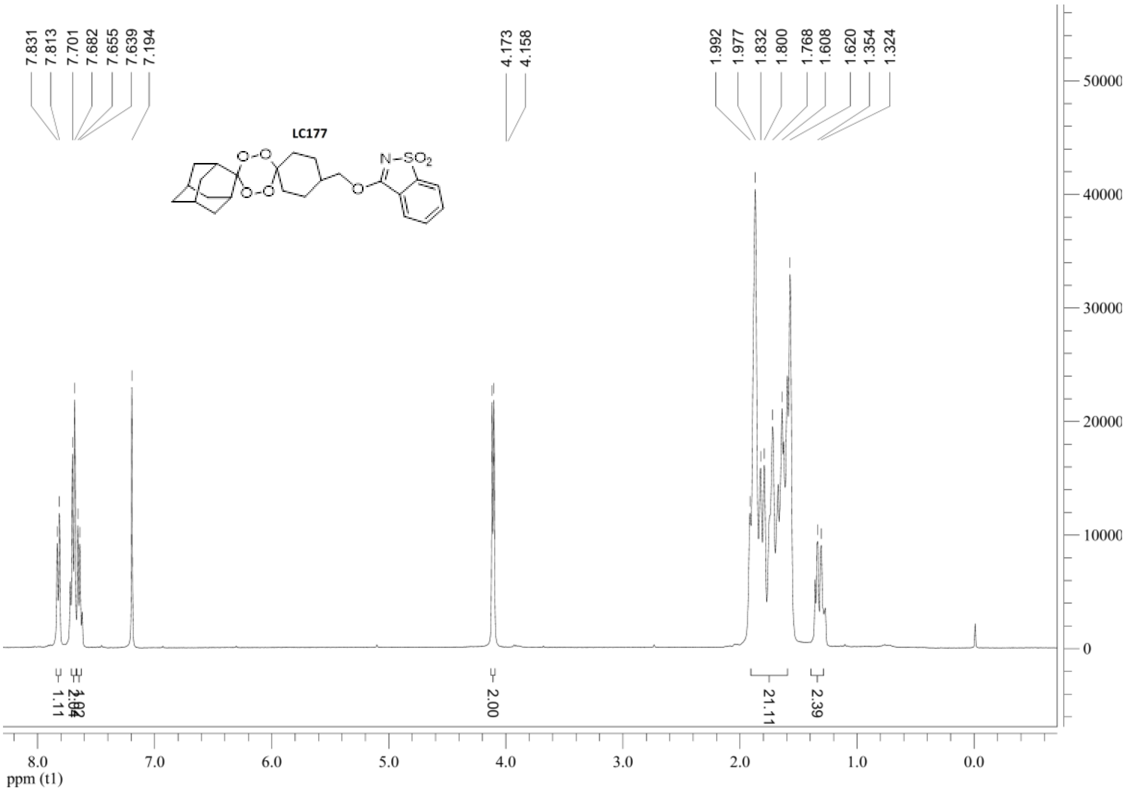

Figure 25. ^{1}H NMR spectrum of compound LC177.

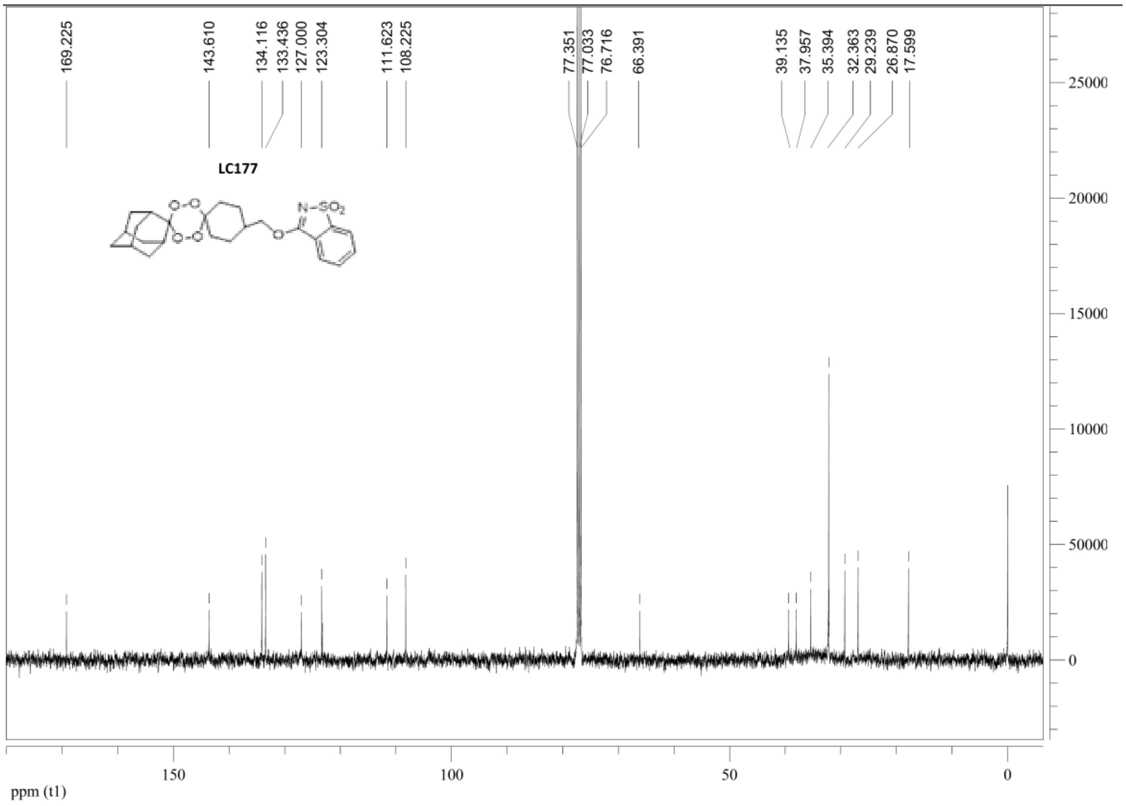

Figure 26. ^{13}C NMR spectrum of compound LC177.

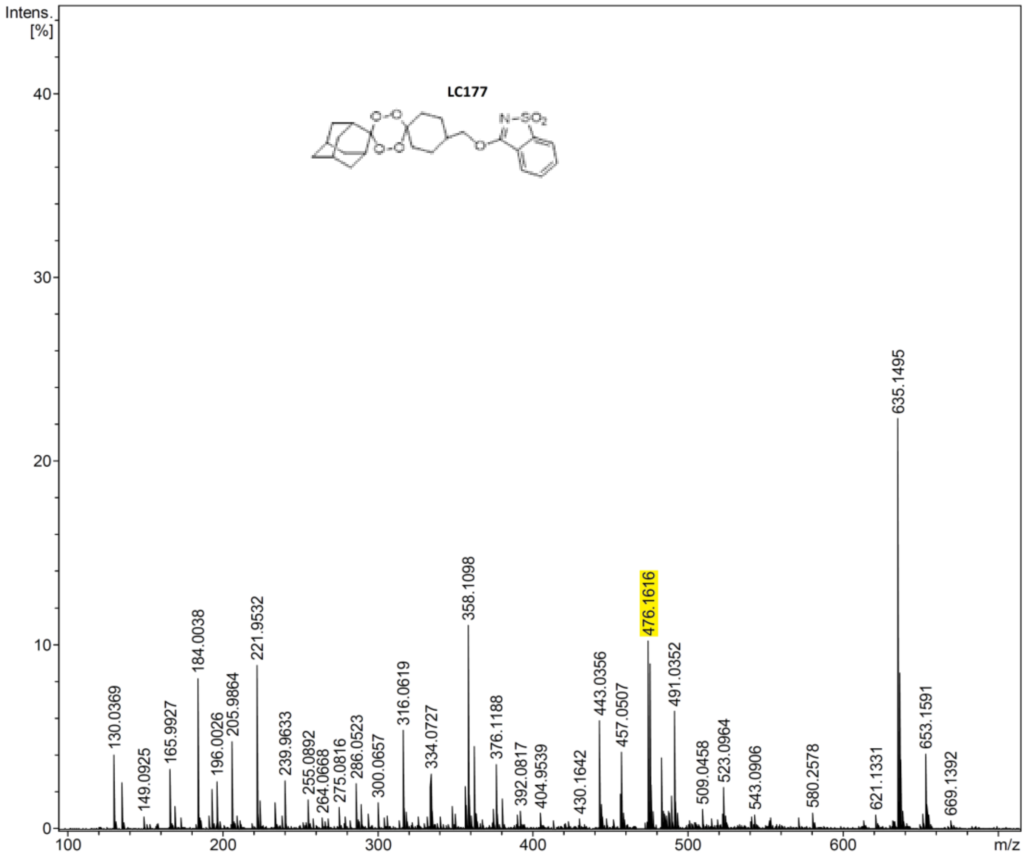

Figure 27. Mass spectrum of compound LC177.

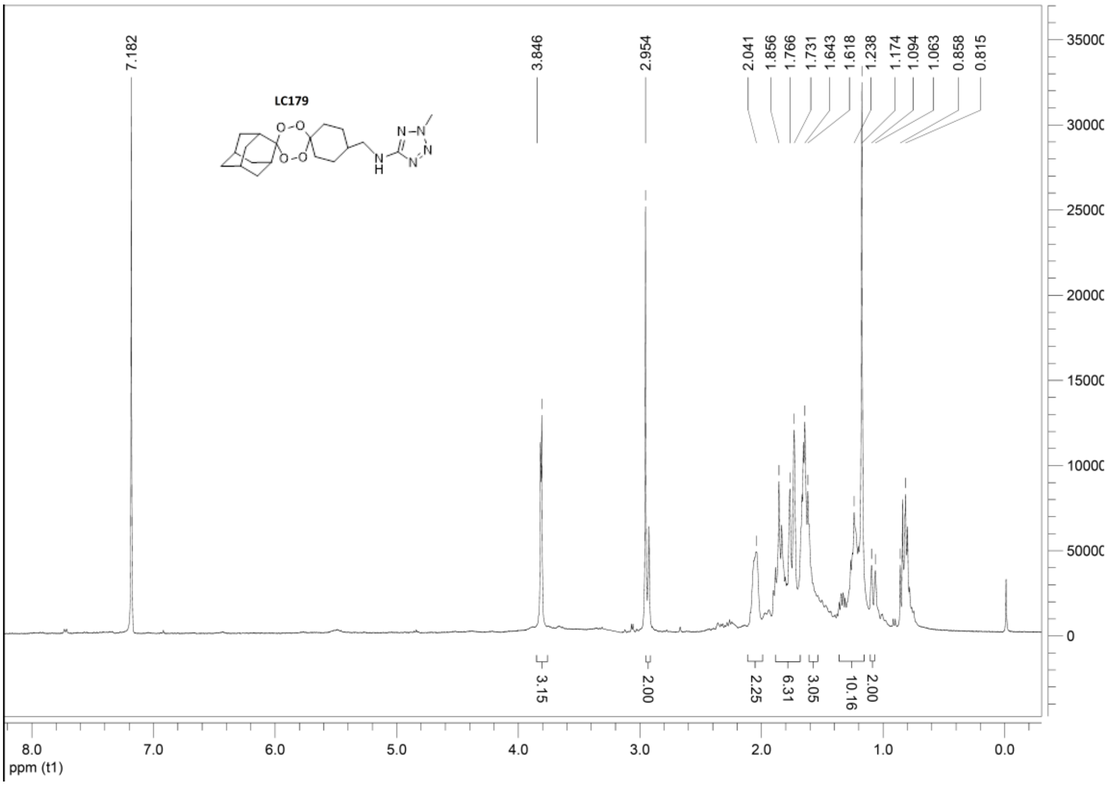

Figure 28. ^{1}H NMR spectrum of compound LC179.

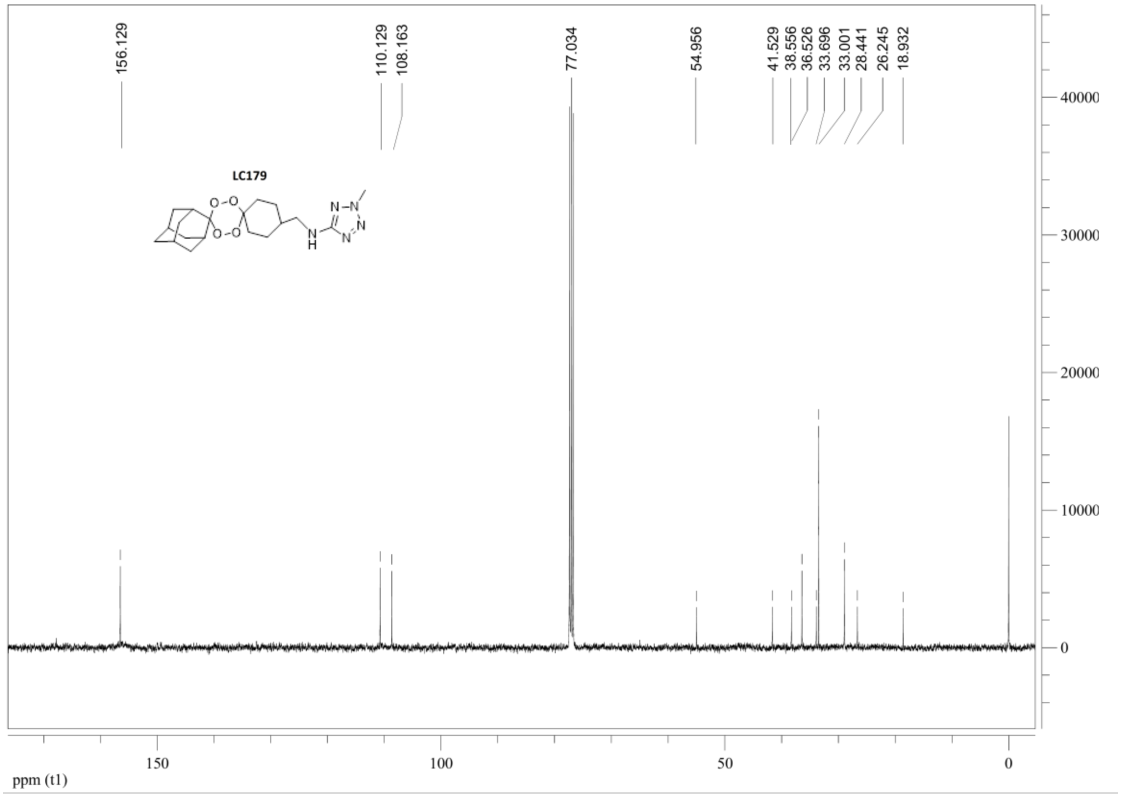

Figure 29. ^{13}C NMR spectrum of compound LC179.

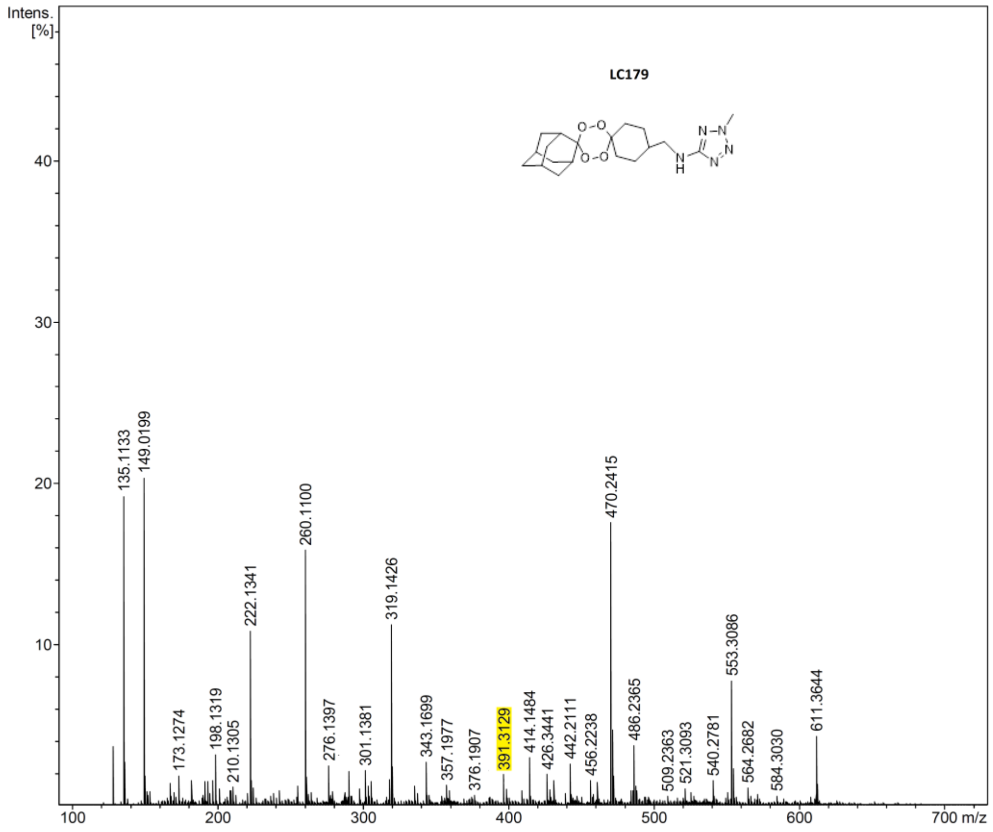

Figure 30. Mass spectrum of compound LC179.

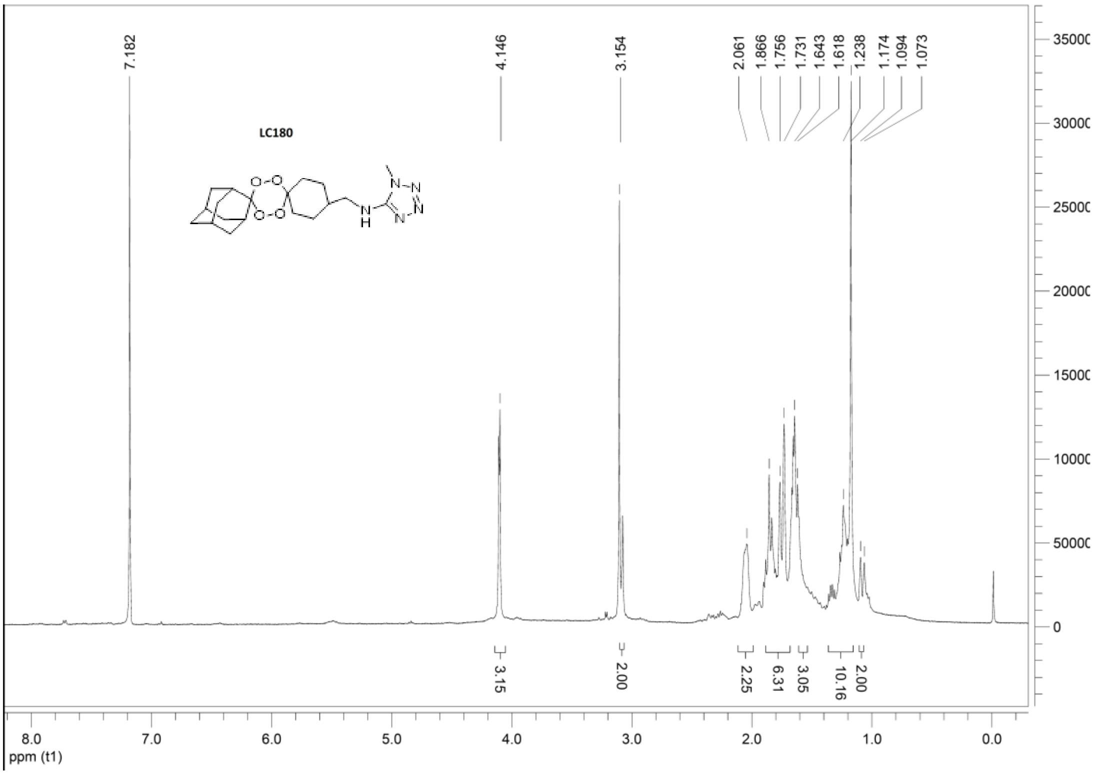

Figure 31. ^{1}H NMR spectrum of compound LC180.

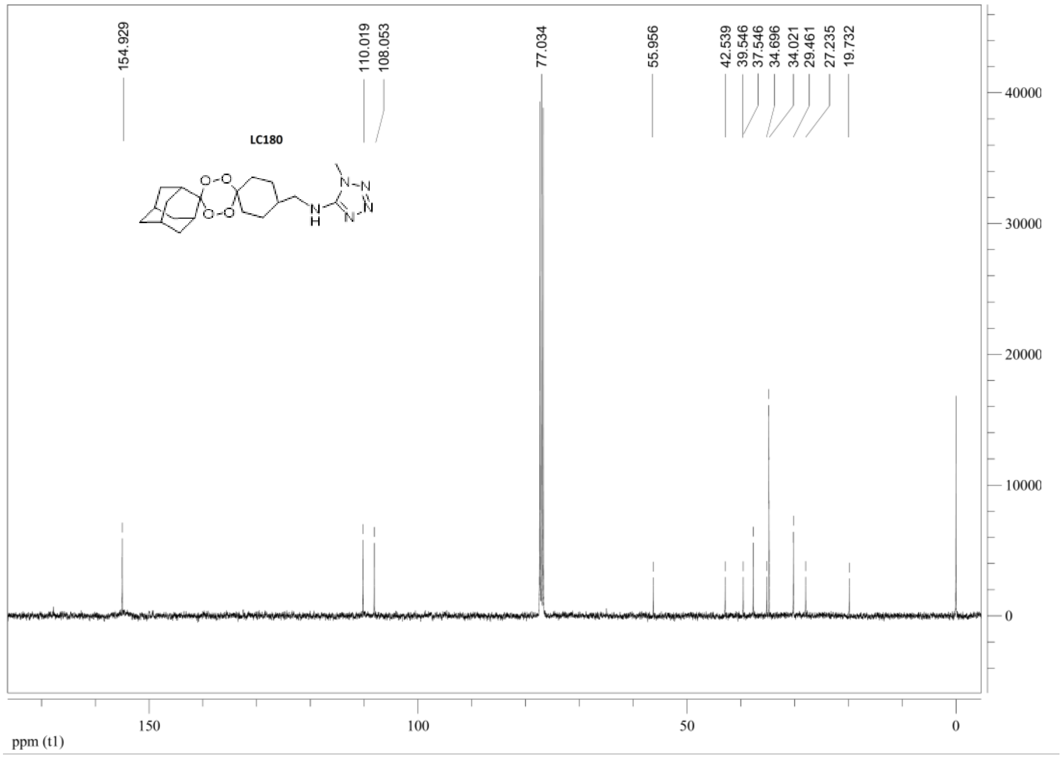

Figure 32. ^{13}C NMR spectrum of compound LC180.

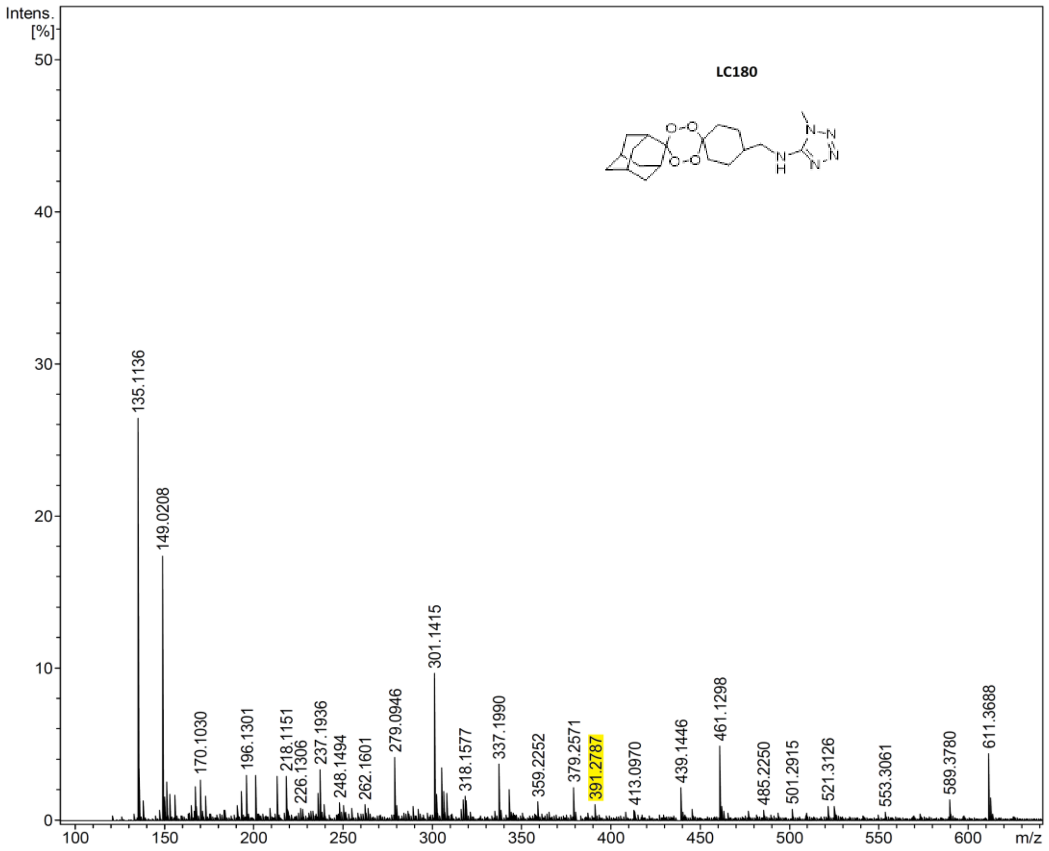

Figure 33. Mass spectrum of compound LC180.

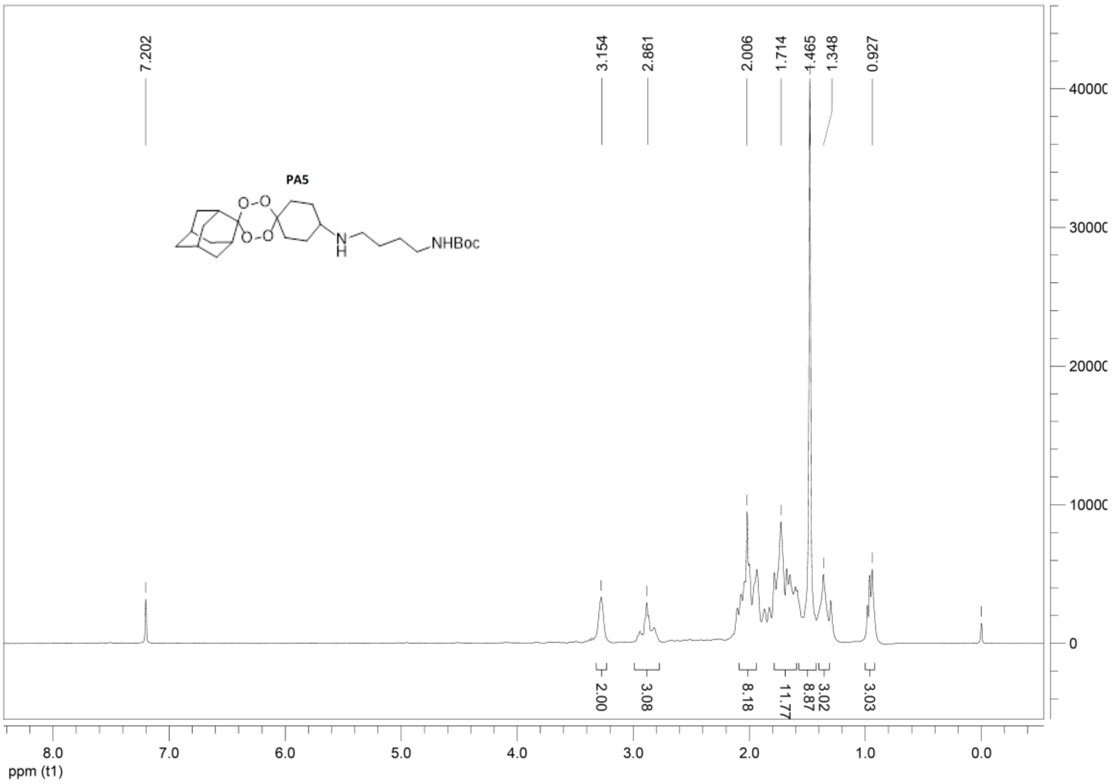

Figure 34. ^{1}H NMR spectrum of compound PA5.

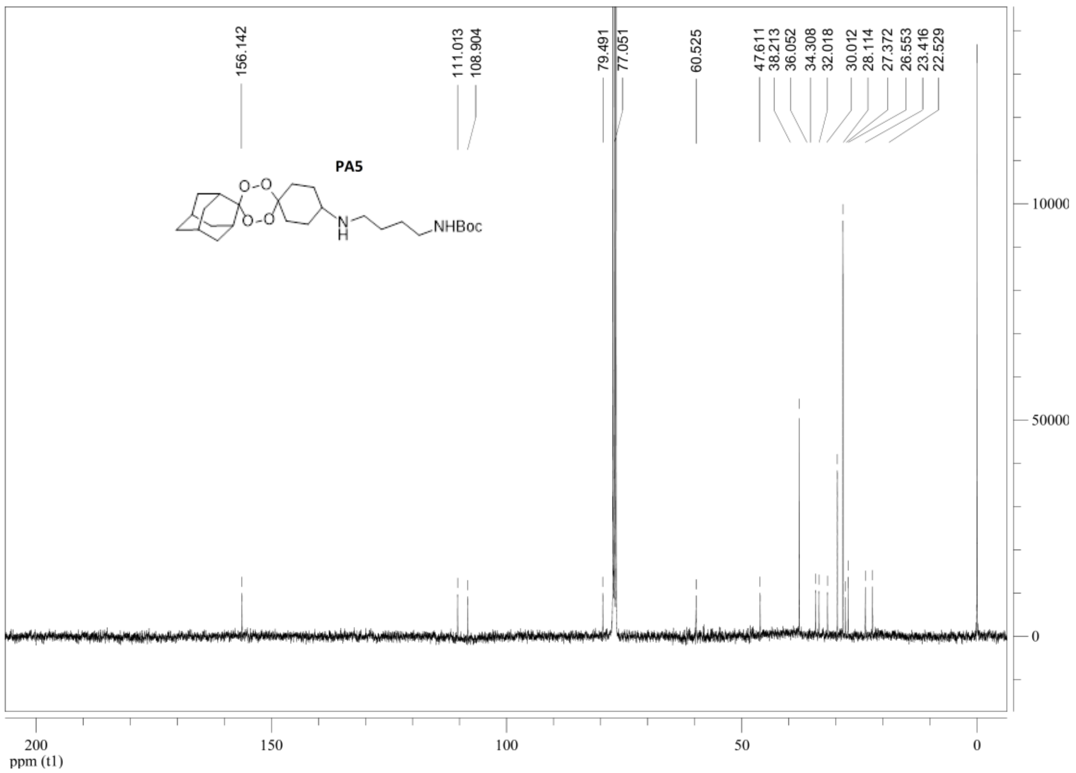

Figure 35. ^{13}C NMR spectrum of compound PA5.

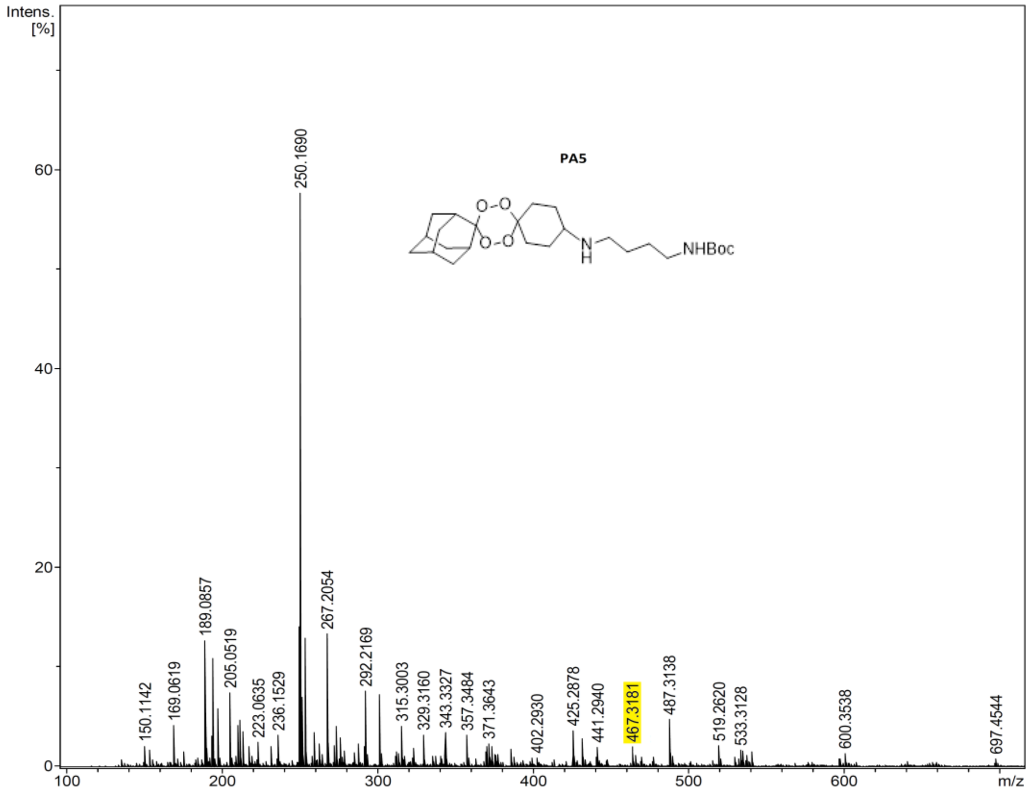

Figure 36. Mass spectrum of compound PA5.

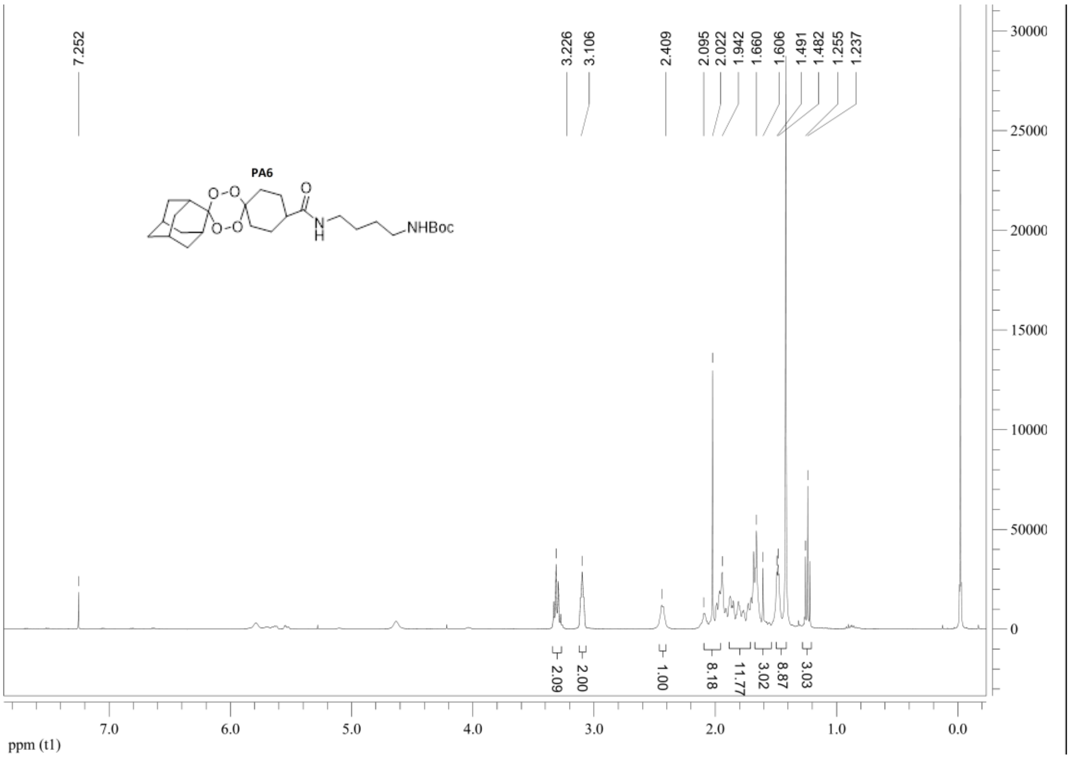

Figure 37. ^{1}H NMR spectrum of compound PA6.

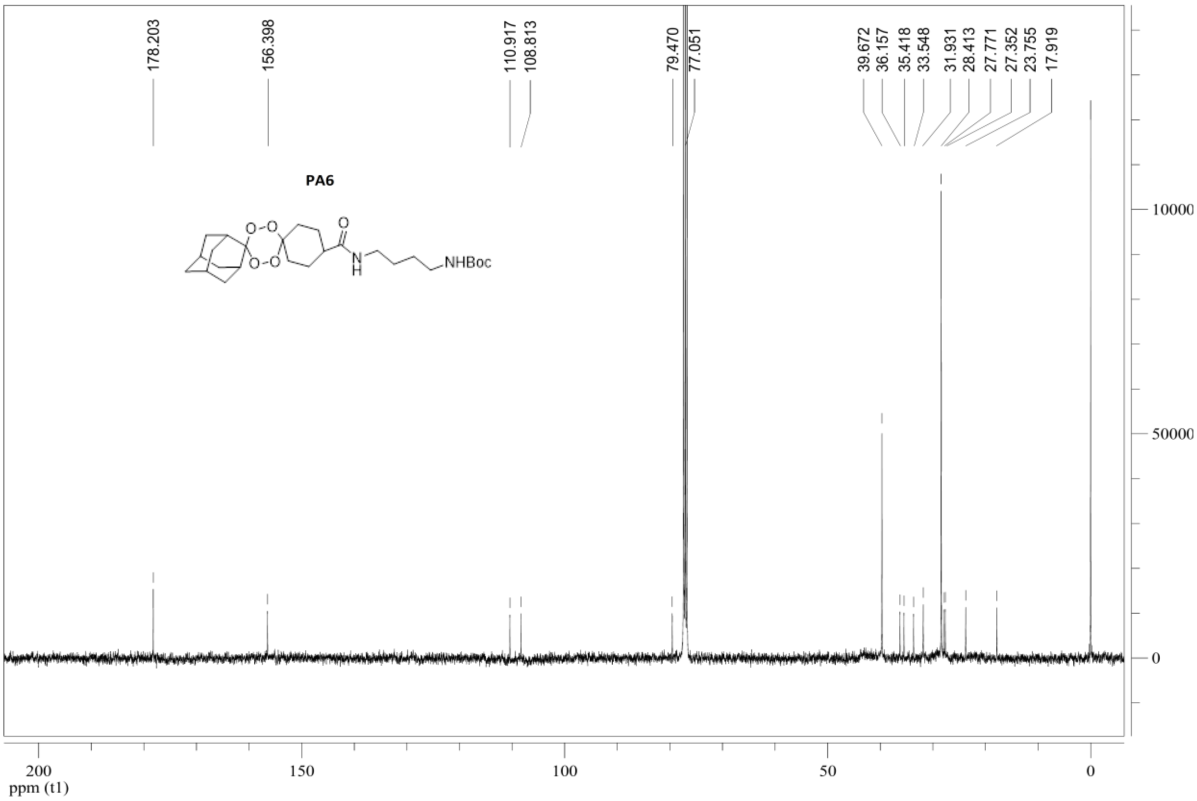

Figure 38. ^{13}C NMR spectrum of compound PA6.

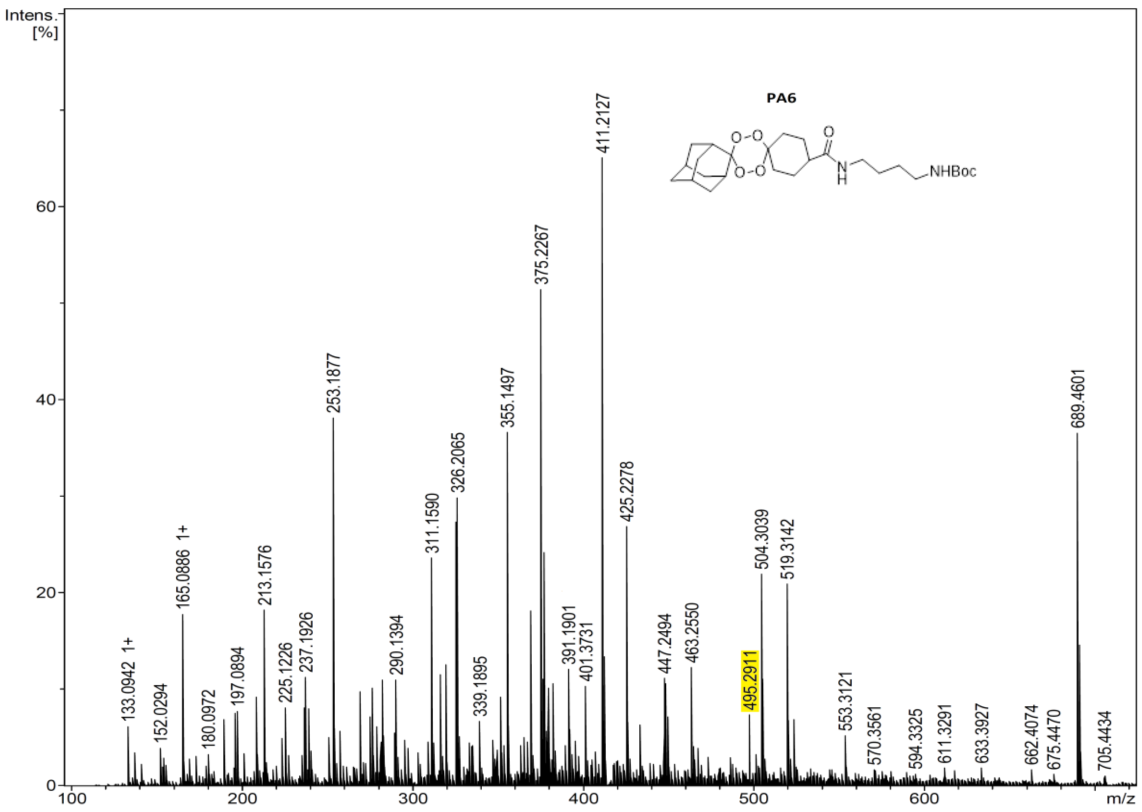

Figure 39. Mass spectrum of compound PA6.

S3. *In vitro* antileishmanial Screening

S3.1. Cell lines and cultures.

The mouse monocyte/macrophage cell line RAW264.7 was maintained in culture in DMEM supplemented with 10% heat-inactivated fetal bovine serum. *Leishmania donovani* (MHOM/ET/67/HU3, also called LV9) was used for *in vitro* experiments. Promastigotes forms were grown in M-199 medium supplemented with 40 mM HEPES, 100 mM adenosine, 0.5 mg/ml haemin, 10% heat-inactivated fœtal bovine serum (FBS) and 50 µg/ml gentamycine at 26 ℃ in a dark environment under an atmosphere of 5% CO_2. Differentiation of promastigotes into axenic amastigotes was achieved by dilution of 106 promastigotes in 5 ml of axenic amastigote medium (15 mM KCl ; 8 mM glucose; 5 mM glutamine, 1 x M-199, 2.5% BBL™ trypticase™ peptone, 4 mM haemin, and 20% Fetal Bovine Serum). The pH was adjusted to pH 5.5. Axenic amastigotes were grown at 37 ℃ in 5% CO_2. All the experiments were performed with parasites in their logarithmic phase of growth.

S3.2. *In vitro* antileishmanial evaluation on intramacrophage amastigotes.

The determination of the cytotoxicity as presented above, was necessary to use the highest drug concentrations to be studied on the intramacrophage amastigote model. The mouse monocyte/macrophage cell line RAW 264.7 was maintained in DMEM supplemented with 10% heat-inactivated fetal bovine serum. RAW 264.7 cells were seeded into a 96-well microtiter plate at a density of $5x10^3$ cells/well in 100 µl of DMEM. After incubation in a 5% CO_2 incubator at 37 ℃ for 24 h, the culture medium was replaced with 100 µl of fresh DMEM containing a suspension of axenic amastigote forms of 10^6 cells/mL. After incubation in a 5% CO_2 incubator at 37 ℃ for 24 h, the culture medium was replaced with 100 µl of fresh DMEM containing the test compounds for a new incubation of 48 h. The viability of the amastigotes into macrophages was then assessed using the SYBR1 Green I (Invitrogen, France) incorporation method. Thus, the medium was removed and the cells were lysed following direct PCR-Cell genotyping without DNA isolation protocol (Euromedex, France). The removed medium replaced by DirectPCR Lysis Reagent (100 µl; Euromedex) before 3 freeze-thaw cycles at room temperature, addition of 50 µg/ml proteinase K, and a final

incubation at 55 °C overnight to allow cell lysis. 10 µl of each cell extract was then added to 40 µl of DirectPCR Lysis reagent containing SYBR Green I (0.05%; Invitrogen). DNA fluorescence was monitored using Mastercycler® realplex (Eppendorf, France). Fluorescence obtained was compared to those from the range obtained with parasite, infected cell and non-infected cell densities. The activity of the compounds was expressed as IC_{50}. Miltefosine was used as the reference drug.

S3.3. *In vitro* antileishmanial evaluation on *Leishmania donovani* axenic amastigotes.

Two fold serial dilutions of the compounds from a maximal concentration of 100 µM were performed in 100 µl of complete medium in 96-well microplates. Triplicates were used for each concentration. A suspension of axenic amastigote forms was prepared to yield 10^7 cells/ml and amastigotes were then added to each well at a density of 10^6/ml in a 200 µl final volume. Cultures were incubated at 37 °C for 72 h in the dark and under a 5% CO_2 atmosphere, then the viability of the amastigotes was assessed using the SYBR1 Green I (Invitrogen, France) incorporation method. Parasite growth was determined by using SYBR1 Green I, a dye with marked fluorescence enhancement upon contact with parasite DNA. Parasites were lysed following Direct PCR-Cell Genotyping without DNA isolation protocol (Euromedex, France). 10 µl of lysed parasite solution of each well was added to 40 µl of PCR-Cell reagent containing the SYBR1 green I in a qPCR plate of 96 wells, and the contents were mixed. Fluorescence was measured with Mastercycler1 ep realplex (Eppendorf, France). Fluorescence obtained was compared to those from the range obtained with different parasite densities. Miltefosine was used as reference compound. The antileishmanial activity was expressed as IC_{50} in µM (concentration of drug inhibiting 50% of the parasite growth, comparatively to the controls treated with the excipient only).

S3.4. Evaluation of compounds cytotoxicity.

Cytotoxicity was evaluated on RAW 264.7 macrophages. RAW 264.7 cells were seeded into a 96-well microtiter plate at a density of 5 x 10^3 cells/well in 100 µl of DMEM. After incubation in a 5% CO_2 incubator at 37 °C for 24 h, the culture medium was replaced with 100 µl of fresh DMEM containing two fold serial dilutions of the test compounds. The starting final concentration was 100 µM. After 48 h incubation time at 37 °C with 5% CO_2, 10

μl of a resazurin solution at 450 μM was added to each well, and the plates were further incubated in the dark for 4 h at 37 °C with 5% CO_2. Cell viability was then monitored by using the resazurin test. In living cells, resazurin is reduced in resorufin and this conversion is monitored by measuring OD570nm (resorufin) and OD600nm (resazurin; Lab systems Multiskan MS). The cytotoxicity of the compounds was expressed as CC_{50} (Cytotoxic Concentration 50%: concentration inhibiting the macrophages metabolism by 50%). Miltefosine was used as the reference drug.

S4. *In vivo* antileishmanial Screening

S4.1. Animal and housing

The animal phase of these studies was conducted at Animex platform of University Paris-Saclay which is committed to the highest standards of laboratory animal welfare and is subject to legislation under the agreement number C 92-019-01. All procedures involving animals were conducted humanely and were performed by or under the direction of trained and experienced personnel. The protocol was reviewed and approved by the Institutional Ethics Committee for Animal Use from Université Paris-Sud (CEEA 26-063/2013) prior to study initiation. The veterinarian was consulted in the overall study design for this study type. These studies were conducted under protocol APAFIS 17860-2018112818574072vl. Swiss CD1 mice (adult females), were obtained from Janvier, Le Genest Saint Isle, France. Animals were individually identified by tail markings and were acclimated to the study environment for 7 days prior to dose administration. Animals were individually housed in suspended wire caging and were kept on a 12h/12h light/dark cycle except when interrupted for study procedures. Average room temperature was regulated in the range 18 to 29 ℃, average relative humidity of 30-70% and an average daily airflow >10 fresh air changes/h.

S4.2. Evaluation of *in vivo* acute toxicity by intraperitoneal route.

Acute toxicity of selected compounds from *in vitro* evaluations was evaluated on 18–20 g female Swiss mice (Élevages Janvier, Le Genest Saint Isle, France) after an intraperitoneal administration at 10 mg/kg under a 0.1 ml volume on 5 mice, comparatively to the control receiving only the excipient (1% methylcellulosis and 0.1% Tween 80). Apparent toxicity signs and death were monitored at 1 min, 15 min, 30 min, 1 h, 4 h, 8 h, and each day until 14 day post-treatment. Animals were weighed before and daily post-treatment. After 14 days, blood samples were collected for a biochemical analysis using an Integra1 800 apparatus (Roche Diagnostic, Paris, France). Current nephrotoxicity was monitored via creatinine assay, and hepatic toxicity via AST (aspartate amino transferase) and ALT (alanine amino transferase) assays. U-Rank test was used for statistical analysis.

S4.3. *In vivo* antileishmanial evaluation

The selected compounds from *in vitro* and *in vivo* acute toxicity analyses were evaluated *in vivo* on the *Leishmania donovani* (MHOM/ET/67/HU3, also called LV9)/BALB/c mice model, comparatively to miltefosine, used as reference drug. Six- to eightweek -old BALB/c mice (Élevages Janvier, Le Genest Saint Isle, France) were infected intravenously on day 1 with 10^7 *L. donovani* amastigotes derived from infected spleen hamsters (*Mesocricetus auratus*) and randomly sorted into groups of 8 mice and two groups of 12 mice, one of them as infected controls treated with excipient, and the other one treated with miltefosine, used as reference drug at the dose of 10 mg/kg. The treatment started one week post-infection, on day 8, and continued for 5 consecutive days until day 12 by oral/intravenous route at the doses of 10 or 20 mg/kg of body weight under a volume of 0.1 ml. At day 16, all groups of mice were autopsied and livers and spleens were weighed. Parasite load in the liver was determined by counting the number of amastigotes/500 liver cells in Giemsa-stained impression smears prepared from the liver and applying Stauber's formula. The slides were counted independently by three persons and the results are expressed as the percentage of reduction of parasite burden comparatively to miltefosine, used as drug of reference. The parasite burden of treatment groups and controls were compared using Student's t test or the Kruskal–Wallis nonparametric analysis of variance test for comparing two groups, or Tukey's/Dunn's multiple comparison test. Significance was established for a P value < 0.05.

All the animal experimental procedures were evaluated and approved by the Institutional Ethics Committee for Animal Use from Université Paris-Saclay (CEEA 26-063/2013).

S.5. References

1. Furniss BS, Hannaford AJ, Smith PWG. 1989. Textbook of Pratical Organic Chemistry. Vogel's. 5th edition London.

2. Brigas AF, Fonseca CSC, Johnstone RAW. 2002. Preparation of 3-chloro-1,2-benzisothiazole 1,1-dioxide (pseudo-saccharyl chloride). *J. Chem. Research (S*). 299-300. doi: 10.3184/030823402103172077

3. Gómez-Zavaglia A, Reva ID, Frija L, Cristiano MLS, Fausto R. Infrared spectrum and UV-induced photochemistry of matrix-isolated 5-methoxy-1-phenyl-*1H*-tetrazole. *Journal of Photochemistry and Photobiology A: Chemistry*. (2006), **180**, 175–183. doi: 10.1016/j.jphotochem.2005.10.012

4. Ismael A, Paixão JA, Fausto R, Cristiano MLS, Molecular structure of nitrogen-linked methyltetrazole-saccharinates. *Journal of Molecular Structure*. (2012), **1023**, 128–142. doi: doi.org/10.1016/j.molstruc.2012.04.018

5. Vennerstrom JL, Dong Y, Chollet J, Matile H. Spiro and dispiro 1,2,4-trioxolane antimalarials. U.S. patent 6,486,199B1 (2002).

6. Lobo L, Cabral LIL, Sena MI, Guerreiro B, Rodrigues AS, De Andrade-Neto VF, Cristiano MLS, Nogueira F. New endoperoxides highly active *in vivo* and *in vitro* against artemisinin-resistant *Plasmodium falciparum*. *Malaria Journal*. (2018), **17**, 1-11. doi: 10.1186/s12936-018-2281-x

7. Terent'ev AO, Kutkin AV, Starikova ZA, Antipin, MY, Ogibin YN, Nikishin GI. New preparation of 1,2,4,5-tetraoxanes. *Synthesis* (2004), **14**, 2356-2366. doi: 10.1055/s-2004-831171

8. Marti F, Chadwick J, Amewu RK, Burrell-Saward H, Srivastava A, Ward SA, Sharma R, Berry N, O'Neill, PM. Second generation analogues of RKA182: synthetic tetraoxanes with outstanding *in vitro* and *in vivo* antimalarial activities. *MedChemComm* (2011), **2,** 661-665. doi: 10.1039/C1MD00102G

9. Wang X, Zhao Q, Vargas M, Dong Y, Sriraghavan K, Keiser J, Vennerstrom JL. The activity of dispiro peroxides against *Fasciola hepatica*. *Bioorg. Med. Chem. Lett.* (2011), **21**, 5320–5323. doi: 10.1016/j.bmcl.2011.07.024
